# Genomic evidence that the live *Chlamydia abortus* vaccine strain 1B is not attenuated and has the potential to cause disease

**DOI:** 10.1016/j.vaccine.2018.05.042

**Published:** 2018-06-14

**Authors:** David Longbottom, Michelle Sait, Morag Livingstone, Karine Laroucau, Konrad Sachse, Simon R. Harris, Nicholas R. Thomson, Helena M.B. Seth-Smith

**Affiliations:** a Moredun Research Institute, Pentlands Science Park, Bush Loan, Edinburgh, Midlothian EH26 0PZ, United Kingdom; b Bacterial Zoonoses Unit, French Agency for Food, Environmental & Occupational Health Safety (Anses), Maisons-Alfort, France; c Friedrich-Loeffler-Institute (Federal Research Institute for Animal Health), Institute of Molecular Pathogenesis, Jena, Germany; d Infection Genomics, Wellcome Sanger Institute, Wellcome Trust Genome Campus, Hinxton, Cambridgeshire CB10 1SA, United Kingdom

**Keywords:** *Chlamydia abortus*, Ovine enzootic abortion, Vaccination, Single nucleotide polymorphisms, Genome analysis

## Abstract

**Background:**

The live, temperature-attenuated vaccine strain 1B of *Chlamydia abortus*, the aetiological agent of ovine enzootic abortion (OEA), has been implicated in cases of vaccine breakdown. The aim of this study was to understand the nature of this attenuation through sequencing of the vaccine parent strain (AB7) and the derived mutant strains 1B and 1H, as well as to clarify the role of the vaccine strain in causing disease through comparative whole genome analysis.

**Methods:**

Whole genome sequencing was performed on: vaccine parent strain AB7; N-methyl-N′-nitro-N-nitrosoguanidine (NTG)-induced temperature attenuated mutant strain 1B grown from the commercial live vaccines Cevac Chlamydia and Enzovax; strain 1H a reverted NTG mutant; and 5 strains isolated from cases of OEA originating from animals from the original vaccine safety trial (2 strains) or from vaccinated ewes or ewes exposed to vaccinated animals (3 strains).

**Results:**

We confirmed that AB7 is in a different lineage from the reference strain S26/3. The genome of vaccine strain 1B contains ten single nucleotide polymorphisms (SNPs) created by the NTG treatment, which are identical to those found in strain 1H. The strains from OEA cases also cluster phylogenetically very tightly with these vaccine strains.

**Conclusions:**

The results show that *C. abortus* vaccine strain 1B has an identical genome sequence to the non-attenuated “reverted mutant” strain 1H. Thus, the protection of the 1B vaccine is unlikely to be due to the NTG induced SNPs and is more likely caused by the administration of high doses of *C. abortus* elementary bodies that stimulate protective immunity. Vaccine-identical strains were also isolated from cases of disease, as well as strains which had acquired 1–3 SNPs, including an animal that had not been vaccinated with either of the commercial live OEA vaccines, indicating that the 1B vaccine strain may be circulating and causing disease.

## Highlights

• 10 SNP differences identified between OEA vaccine strain 1B and parent strain AB7.
• Genomic sequences of OEA vaccine strain 1B and reverted mutant strain 1H identical.
• No mutations in vaccine strain likely to alter its propensity to cause disease.
• No genomic evidence of any attenuation in OEA vaccine strain 1B.
• Protection of 1B vaccine strain unlikely to be due to any chemically induced SNPs.

## 1 Introduction

The Gram-negative, obligate intracellular bacterium *Chlamydia abortus* (formerly *Chlamydophila abortus*) is one of the most common causes of infectious abortion in small ruminants worldwide [1], [2]. Vaccines targeting the disease (known as ovine enzootic abortion (OEA) or enzootic abortion of ewes (EAE)) have been developed commercially [3], including two live attenuated vaccines (Enzovax®, MSD Animal Health; Cevac® Chlamydia, Ceva Animal Health Ltd), which are based on an attenuated mutant strain of *C. abortus* (strain 1B) that was generated following N-methyl-N′-nitro-N-nitrosoguanidine (NTG) mutagenesis of a wild-type field strain (AB7) isolated from an aborted lamb [4]. Mutagenesis of AB7 produced two temperature-sensitive mutant strains, 1B and 1H, that were characterized as having a reduced growth rate at 39.5 °C and increased thermolability at 51 °C resulting in a reduced infectivity and ability to induce death *in utero* when compared to parent strain AB7 [5], [6]. Strain 1H was subsequently found to be unstable and reverted to virulence but 1B was stable after multiple passages *in vitro* and *in vivo* [7] and could protect against *C. abortus* infection, reinfection and shedding [6], [8].

To understand the basis of the attenuation, strain 1B, the reverted mutant strain 1H and parent strain AB7 were sequenced using NimbleGen technology [9]. Comparative genome analysis of AB7 with the *C. abortus* reference sequence strain S26/3 identified 591 single-nucleotide polymorphisms (SNPs), while only 22 SNPs were identified between AB7 and 1B, 20 of which were also present in 1H. Analysis of the 22 mutations led to the conclusion that the temperature-sensitive phenotype of 1B resulted from disrupted metabolic activity, altered pyruvate kinase expression and/or the alteration of the function of membrane proteins [9].

In order to further understand the nature of the attenuation leading to the temperature-sensitive phenotype of 1B we have performed full genome resequencing of the two mutant strains 1B and 1H and parent strain AB7 using Illumina technology. Vaccine strain 1B has also been uniquely detected in the placentas of vaccinated sheep that have aborted as a result of OEA in comparable numbers to those found in wild-type infections, implicating the vaccine-derived strain as a probable cause of disease in some vaccinated animals [10], [11], [12], [13]. Therefore, we additionally aimed to clarify this likely causal role of the vaccine strain in cases of OEA in vaccinated animals through whole genome comparative analysis.

## 2 Material and methods

### *2.1* *Chlamydia abortus* strains, propagation and preparation of genomic DNA

Nine *C. abortus* strains were selected for genome sequencing: reverted NTG-mutant strain 1H; virulent field strain AB7, isolated in France from an aborted lamb [4], from which 1B and 1H were derived [5]; two different preparations of the NTG-mutant strain 1B prepared from the commercial vaccines Enzovax® (MSD Animal Health, UK; referred to as 1B-Enzovax) and Cevac Chlamydia® (Ceva Animal Heath Ltd, UK; referred to as 1B-Cevac); two strains (6012 and 6181) isolated in Scotland in 1994 from vaccinated ewes that subsequently aborted during the original Enzovax field safety trial [11]; and three field strains isolated from the placentas of vaccinated ewes that aborted (strain 10DC0084 from Germany [14]) and 11-669_5380/2 from France [15]) and from the vaginal swab of an unvaccinated aborted ewe that had extensive contact with an OEA vaccinated herd (strain AB15 from France [10]).

Strains were propagated and infected cells harvested to purify chlamydial elementary bodies for genomic DNA extraction, as described previously [16] or by using a Wizard DNA extraction kit (Promega, UK).

### 2.2 Genome sequencing, mapping, assembly and analysis

*C. abortus* AB7, 1B-Cevac, 1B-Enzovax, 1H, 11-669_5380/2, 10DC0084, AB15, 6012 and 6181 genomes were sequenced using the Illumina HiSeq platform with 75-bp paired end reads resulting in a mean genome coverage of 135, 300, 14, 208, 40, 273, 169, 10 and 10x, respectively, after mapping against reference strain S26/3 [16] using SMALT (http://www.sanger.ac.uk/science/tools/smalt-0), with a minimum identity threshold for mapping of 80%. For 1B-Cevac, reads were assembled using Velvet v1.0.12 [17] with manual improvement to produce a single contig. The genomes of the other 8 strains were derived through mapping of sequence reads against the 1B-Cevac and S26/3 [16], [18] reference genomes using SMALT. SNPs were called using GATK with indel realignment and were manually checked against mapped reads with even the lower coverage samples providing sufficient data for confident base calls.

Annotation was transferred from strain S26/3 [16] to the genomes of AB7 and 1B using AnnotateBacteria [https://github.com/sanger-pathogens/Bio-AutomatedAnnotation/blob/master/lib/Bio/AutomatedAnnotation/CommandLine/AnnotateBacteria.pm], as previously described [19], and manually curated using Artemis [20]. Comparative genome analysis was performed using the Artemis Comparison Tool (ACT) [21]. The AB7, 1B and 1H genomes were manually curated for the presence of pseudogenes, which were defined as having one or more mutations (frameshift, premature stop codon) that would ablate expression.

### 2.3 PCR SNP analysis

The 22 SNP differences between strains AB7, 1B and 1H that were identified previously [9], as well as new SNPs identified in this study, were additionally verified by PCR in all 3 strains. PCR was performed in 50 µl reactions containing 1 × Biomix (Bioline, London, UK), 10 pmol each primer (Table S1) and 50 ng genomic DNA. PCR cycle conditions comprised: denaturation at 94 °C for 2 min; 35 cycles of denaturation at 94 °C for 15 s, primer annealing at 52–60 °C (depending on primer Tm) for 15 s and extension at 72 °C for 15 s; and a final extension step at 72 °C for 2 min. PCR products were purified using a QIAquick PCR Purification Kit (QIAGEN Ltd., Manchester, UK), according to manufacturer’s instructions and sequenced by Eurofins Genomics (Ebersberg, Germany). Amplicon sequences were aligned against the S26/3, AB7 and 1B genome sequences using MegAlign Pro (DNASTAR Lasergene suite; https://www.dnastar.com/t-allproducts.aspx) to verify the presence or absence of SNPs.

### 2.4 Nucleotide sequence accession numbers

Genome sequences of AB7, 1B-Cevac and 1H have been deposited in EMBL under accession numbers LN554882, LN589721 and LN554883, respectively. Read data for AB7, 1B-Cevac, 1B-Enzovax, 1H, 6012, 6181, 10DC0084, 11-669_5380/2 and AB15 have been deposited in the European Nucleotide Archive (ENA, http://www.ebi.ac.uk/ena/) under accessions ERS200120, ERS200134, ERS067056, ERS200121, ERS200136, ERS200137, ERS067060, ERS200127 and ERS200131, respectively.

## 3 Results

### 3.1 Comparative analysis of *C. abortus* vaccine parent strain AB7 to strain S26/3

**Fig. 1:**
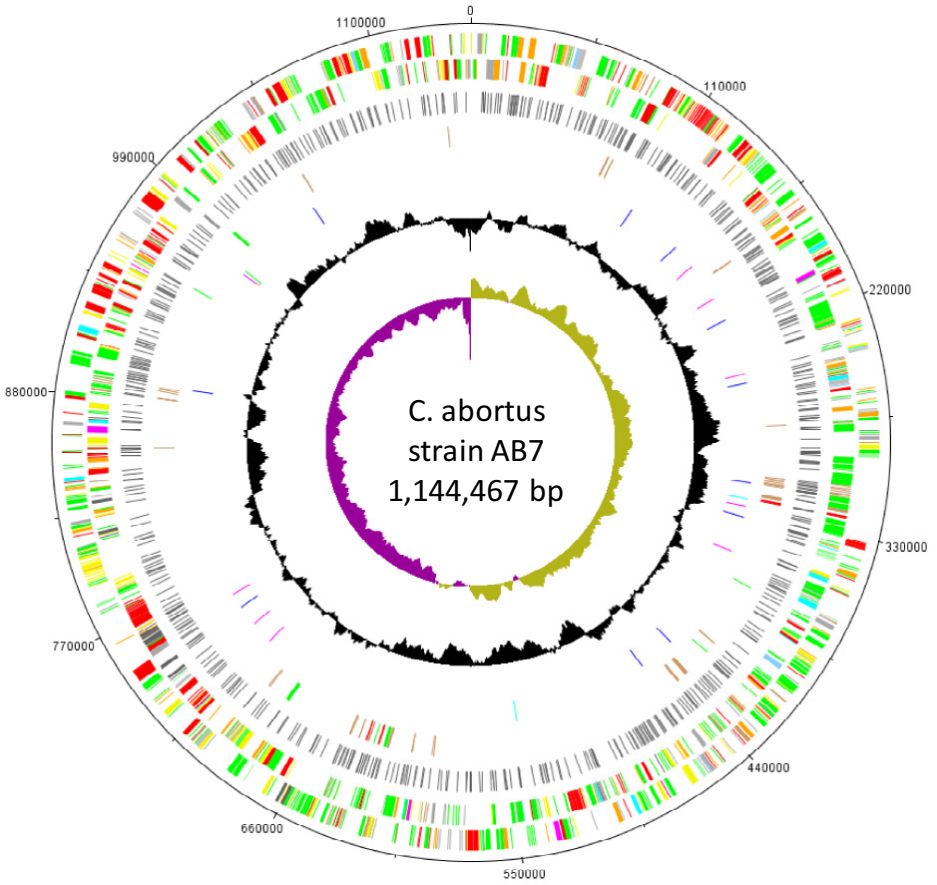
Circular representation and genome comparison of *C. abortus* strains AB7, S26/3 and 1B/1H. The outer scale shows the size in base pairs. From the outside in: tracks 1 and 2 show the position of genes transcribed in a clockwise and anticlockwise direction, respectively. CDSs in tracks 1 and 2 are colour coded according to the function of their gene products: membrane or surface structures (dark green); central or intermediary metabolism (yellow); degradation of macromolecules (cyan); information transfer/cell division (red); degradation of small molecules (purple); regulators (pale blue); pathogenicity or adaptation (dark blue); energy metabolism (black); conserved hypothetical (orange); unknown (pale green); pseudogenes (brown). Track 3, SNP differences between AB7 and S26/3. Track 4, pseudogene similarities and differences between AB7 and S26/3: shared pseudogenes (brown); S26/3-specific pseudogenes (dark green); AB7-specific pseudogenes (red). Track 5, SNPs present in 1B relative to AB7: Burall et al. [9] SNPs confirmed (magenta) or not identified (dark blue) in this study; Burall et al. [9] SNPs in 1B relative to 1H which were not found in this study (cyan); unique SNP found in this study (dark green). Track 6, G + C content plot (in a 10 kb window); track 7, GC skew plot ([G − C]/[G + C] (in a 10 kb window). (For interpretation of the references to color in this figure legend, the reader is referred to the web version of this article.)

The genome of strain AB7 was determined to be 1,144,467 bp with a G+C content of 39.86%, containing 960 CDSs, a single rRNA operon and 38 tRNA genes. After careful read pair analysis, *pmp12G* was found to be identical to *pmp17G*, as in S26/3 [16]. AB7 was found to have 604 SNPs compared to S26/3, affecting 371 CDSs and 75 predicted intergenic regions, plus 59 indels (Table S1). SNPs and indels were distributed evenly throughout the AB7 genome (Fig. 1; Table S2). A total of 283 non-synonymous mutations plus ten indels, affecting 237 CDSs were identified. Twenty-one of the 30 pseudogenes present in S26/3 [16] were found to have SNPs and indels in AB7 (Table S2), which either did not affect their pseudogene status (n = 14) or produced intact CDSs (n = 7).

Overall 26 pseudogenes were identified in AB7, of which three were unique in comparison with S26/3 (Table S3). These three unique pseudogene differences resulted from single frame-shift mutations present in the homopolymeric nucleotide tracts of *pmp13G* (AB7_3131), a hypothetical protein gene (AB7_6101) and a conserved hypothetical membrane protein gene (AB7_6181), and thus possibly subject to phase variable expression [16]. Of the remaining 23 pseudogenes that are present in both AB7 and S26/3, three also have single frame-shift mutations in homopolymeric nucleotide tracts (CAB279/AB7_3121, CAB383A/AB7_4301 and CAB516/AB7_5781), while the others have single or multiple frame-shifts/premature stop codons (n = 16) and/or lack a translational start site (n = 4) (Table S3).

### 3.2 Comparative analysis of the NTG-derived mutants 1B and 1H with their parent strain AB7

The genomes of 1B-Cevac and 1B-Enzovax were determined to be identical. We identified ten SNP differences between the genomes of 1B and AB7, which included nine of the 22 previously identified SNPs [9], and an additional SNP (within CAB843/AB7_9301/Cevac_9301) not previously identified (Table 1). Of the other 13 previously identified SNPs [9], we found 11 to be also present in AB7 and thus identical between AB7 and 1B, while the remaining two were found to be identical in S26/3, AB7 and 1B (SNPs in CAB281/AB7_3131/Cevac_3131 and CAB469/AB7_5251/Cevac_5251) and thus not SNPs in any of the studied genomes (Table 1). Due to the more advanced technology and high coverage used in this study, in conjunction with the thorough manual confirmation of SNPs (Fig. S1) and PCR analysis of all SNP differences between 1B, 1H and AB7 (Table S1), we have very high confidence in the genomic data presented.

**Table 1:** SNPs identified in *C. abortus* mutant strains 1B/1H relative to vaccine parent strain AB7 and UK reference strain S26/3.

Genomic position^a^		CDS^b^		Protein product	SNPs: Burall et al. (2009)			SNPs: this study				
S26/3	AB7 & 1B	S26/3	AB7 & 1B		S26/3 & AB7	1H	1B	S26/3	AB7	1H & 1B	Mutation class^c^	Amino acid change^d^
110655	110648	096	1071	putative 50S ribosomal protein l2	C	A	A	C	A	A	–	–
147575	147568	139	1531	putative lipoprotein	C	T	T	C	T	T	–	–
164031	164286	153	1701	putative sigma-54 dependent response regulator	C	T	T	C	C	T	Non	E → K
189518	189504	175	1951	putative protein export protein	G	A	A	G	G	A	Non	V → I
205074	205059	n/a^e^	n/a^e^	n/a^e^	C	T	T	C	T	T	–	–
241667	241651	220	2471	putative serine hydroxymethyltransferase	G	A	A	G	G	A	Non	D → N
247417	247401	227	2531	conserved hypothetical protein	G	A	A	G	A	A	–	–
312724	312707	273	3051	Pmp9G (pseudogene)	C	A	A	C	A	A	–	–
323028	323012	281	3131	Pmp13G	G	G	A	G	G	G	–	–
328034	328018	283	3171	pmp15G	C	T	T	C	C	T	Non	W → *^f^
335181	335165	n/a^e^	n/a^e^	n/a^e^	T	C	C	T	C	C	–	–
358346	358327	308	3441	putative lipoprotein	C	T	T	C	C	T	Non	E → K
429384	429384	373	4181	putative inner membrane protein	T	C	C	T	C	C	–	–
453290	453312	394	4431	putative glycosyl hydrolase	A	C	C	A	C	C	–	–
542479	542469	469	5251	recR	A	A	G	A	A	A	–	–
716144	716136	622	6911	methionyl-tRNA synthetase	C	T	T	C	C	T	Non	E → K
731338	731329	636	7071	putative phosphate starvation-inducible protein	C	T	T	C	C	T	Non	G → D
744408	744400	n/a^e^	n/a^e^	n/a^e^	C	T	T	C	T	T	–	–
754284	754282	648	7201	valyl-tRNA synthetase	G	A	A	G	G	A	Non	P → S
891683	891735	772	8531	putative TMH-family membrane protein	A	T	T	A	T	T	–	–
974264	974300	842	9291	putative alanyl-tRNA synthetase	G	A	A	G	G	A	Syn	–
976325	976361	843	9301	putative transcription-repair coupling factor	G	G	G	G	G	A	Non	G → S
1036695	1036784	887	9791	trigger factor	A	C	C	A	C	C	–	–

a Genomic position in the S26/3 (CR848038), AB7 (LN554882) and 1B-Cevac (LN589721) genomes.

b CDS, coding sequence. Gene number of coding sequence containing the specific SNP in S26/3 (designated CABxxx), AB7 (AB7_xxxx) and 1B-Cevac (CEVAC_xxxx).

c Non-synonymous (Non) or synonymous (Syn) codon change resulting from SNPs or intergenic (Int) location of SNPs in 1H and 1B relative to AB7.

d Amino acid change resulting from SNPs in AB7, 1H and 1B relative to S26/3 or 1H and 1B relative to S26/3 and AB7.

e n/a, not applicable as in intergenic region.

f *, stop codon.

The ten confirmed SNPs differentiating 1B from AB7 are found in genes encoding a sigma-54-dependent response regulator (Cevac_1701/CAB096), export protein (Cevac_1951/CAB175), serine hydroxymethyltransferase (Cevac_2471/CAB220), Pmp15G (Cevac_3171/CAB283), lipoprotein (Cevac_3441/CAB308), methionyl-tRNA synthetase (Cevac_6911/CAB622), phosphate starvation-inducible protein (Cevac_7071/CAB636), valyl-tRNA synthetase (Cevac_7201/CAB648), alanyl-tRNA synthetase (Cevac_9291/CAB842) and a putative transcription-repair coupling factor (Cevac_9301/CAB843; the newly identified SNP) (Table 1). All these mutations, with the exception of the SNP within Cevac_9291/CAB842, are non-synonymous. The SNP in *pmp15G* (Cevac_3171/CAB283) results in a premature stop codon and truncation of the protein product. No indels were identified. All SNPs are annotated in the deposited genome of 1B-Cevac. The two SNPs that previously differentiated the non-attenuated reverted mutant strain 1H from 1B (CAB281 and CAB469 in [9]; Cevac_3131 and Cevac_5251 in Table 1 in this study) were not present, revealing the genomes of 1H and 1B to be identical.

### 3.3 Genome sequencing of strains isolated from cases of OEA

**Fig. 2:**
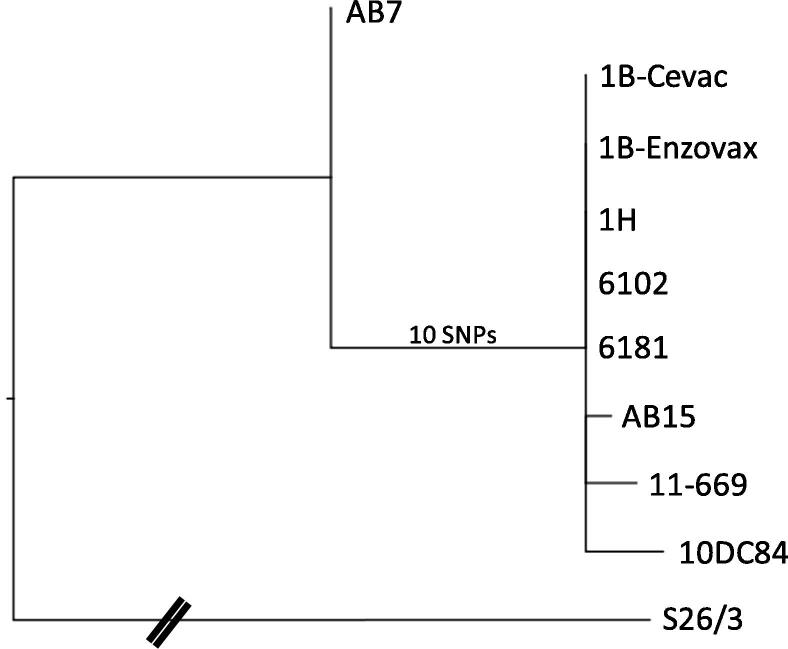
Phylogenetic tree showing the relationship of strains within the vaccine strain clade using reference strain S26/3 as the outgroup. For clarity, the distance to S26/3 is not representative (as indicated by the dashed lines) and represents 604 SNPs from AB7. Ten SNPs separate AB7 from 1B-Cevac/1B-Enzovax with AB15, 11_669_5380/2 and 10DC0084 containing an additional 1, 2 and 3 SNPs, respectively.

The strains 6012 and 6181, isolated from the aborted placentas of sheep vaccinated with Enzovax during the original vaccine safety trials, were found to have identical genome sequences to strain 1B (Fig. 2). Field strains 11_669_5380/2 and 10DC0084, isolated from animals vaccinated with 1B-Enzovax or 1B-Cevac and aborted as a result of OEA, were found to have two or three SNPs in addition to the ten unique SNPs, therefore being clearly descended from the NTG-derived strains (Fig. 2, Table 2). The two SNPs in 11_669_5380/2 consist of a G → A SNP in the intergenic region upstream of Cevac_1941 and a T → G SNP resulting in a Cys → Gly substitution in a hypothetical protein (Cevac_6631). The three additional SNPs in 10DC0084 consist of an A → T SNP resulting in a non-synonymous substitution in a CDS encoding a phosphate permease (Cevac_0681), a C → T SNP resulting in an Ala → Val substitution in a hypothetical protein (Cevac_1301) and a C → T SNP resulting in a synonymous substitution in a 1,4 alpha-glucan branching enzyme (Cevac_2911). Strain AB15, from an unvaccinated animal that had extensive contact with a flock vaccinated with the live 1B *C. abortus* vaccine strain, was also found to cluster with the 1B vaccine strain, with one further unique T → G SNP resulting in a Ser → Ala substitution in a putative alcohol phosphatidyltransferase (Cevac_1381).

**Table 2:** Additional SNPs present in *C. abortus* field strains isolated from the placentas of sheep that have aborted as a result of OEA.

Strain	Genomic position^a^	CDS	Predicted function	SNP	Amino acid change
10DC0084	75867	Cevac_0681	Phosphate permease	A → T	L → F
	123293	Cevac_1301	Conserved hypothetical protein	C → T	A → V
	288668	Cevac_2911	1,4-alpha-glucan branching enzyme	C → T	H
11_669_5380/2	185776	Intergenic		G → A	n/a^b^
	681841	Cevac_6631	Conserved hypothetical protein	T → G	C → G
AB15	130861	Cevac_1381	Alcohol phosphatidyltransferase	T → G	S → A

a Genomic position on the 1B-Cevac genome (LN589721).

b n/a, not applicable.

## 4 Discussion

Comparative genomic analysis of the *C. abortus* vaccine parent strain AB7 with UK reference strain S26/3 revealed very similar results to the Burall et al. [9] study, with an additional 13 SNPs affecting an additional eight CDSs in AB7. We did note a discrepancy in the number of indels present, with an additional 51 (59 in total) identified in our study. The majority of these indels (n = 49) are present in non-protein coding intergenic regions, while those present in CDSs (n = 10) are predicted to have little effect on protein coding, either adding a few additional amino acids or affecting the last few C-terminal amino acids. Burall et al. [9] noted that three of the 14 genes they observed with more than two protein-altering non-synonymous mutations were *pmp* genes, consistent with their characterization as a highly polymorphic gene family. In our study we identified 15 genes with three or more non-synonymous mutations, of which four are *pmp* genes (CAB200/AB7_2251, CAB270/AB7_3031, CAB273/AB7_3051 and CAB776/AB7_8571), and two of these (CAB270/AB7_3031 and CAB273/AB7_3051) are pseudogenes with additional frame-shifts in homopolymeric tracts, suggesting they are possibly subject to phase-variable expression. Again, this is consistent with them being a highly polymorphic gene family and having a role in disease pathogenesis [16].

Comparison of the parent (AB7) and mutant (1B and 1H) vaccine strains, however, revealed notable differences with the Burall et al. [9] study, where 22 SNP differences were identified between the mutant strains and AB7 using Nimblegen resequencing arrays. Our study, using a more accurate high-throughput sequencing approach and genome coverage of 300×, has identified only ten, including eight of the originally identified non-synonymous mutations plus an additional previously unidentified non-synonymous mutation in a transcription-repair coupling factor protein (Cevac_9301), as well as a synonymous mutation in an alanyl-tRNA synthetase (Cevac_9291). Of the 13 mutations originally identified [9] that were found to be absent in our study by sequencing and PCR analysis, 12 were synonymous (n = 9) or in intergenic regions (n = 3), while the thirteenth was a non-synonymous mutation in *pmp9G* (Cevac_3051), which although ablating a premature stop codon in the corresponding protein product did not affect the pseudogene status of this gene. Intriguingly, the two previously identified differences between the vaccine mutant strain 1B and reverted mutant strain 1H [9] in *pmp13G* (Cevac_3131/ CAB281) and *recR* (Cevac_5251/ CAB469) were not present in our study, with the same nucleotides present in all four strains (S26/3, AB7, 1H and 1B). This agrees with a previous study that had already revealed the absence of one of these SNPs (Cevac_3131/CAB281) [11]. The fact that 1B and 1H are completely identical raises important questions concerning the nature of the temperature-sensitive phenotype of the 1B strain. Indeed, Burall et al. [9] made little comment or discussion with regard to the differences between 1B and reverted mutant strain 1H, other than suggesting that 1B is derived from 1H and that the two identified silent mutations could affect a regulatory sequence and protein folding through differences in codon usage; however, as we now know the two strains to be genomically identical these possibilities are no longer relevant. Irrespective of this 1H versus 1B issue, the authors did go on to suggest that the attenuated phenotype of 1B results from multiple mutations affecting the Pho regulon, altered pyruvate kinase activity (resulting from a mutation affecting promoter function for CAB645), and membrane proteins Pmp15G and a lipoprotein. While these mutations have been validated in our study, with the exception of the mutation affecting CAB645, we think that there is a more likely explanation as to why the live vaccines protect sheep from infection, but yet appear to have the capacity to cause infection.

In previous studies we have provided molecular (PCR-RFLP and quantitative PCR) evidence suggesting that the commercial live vaccines have the capacity to cause abortion in some vaccinated animals [11], [13], [22]. In this study we provide genomic evidence that such strains isolated from cases of OEA contain the same SNPs as those present in the 1B vaccinal strain, based on isolates collected from vaccinated aborted ewes during the original vaccine safety trials conducted in the UK in the 1990s, or prepared very recently from the two purchased commercial vaccines. This suggests that the vaccine strain has remained unchanged over the last 20 years of use at least and so there are no additional mutations that could be linked to an altered propensity to cause disease. This also provides strong evidence that the strains present in these vaccinated and aborted animals are indeed the vaccinal strain. If this is the case then this means, as previously suggested [22], that this live vaccine has likely always had the capacity to cause disease, but that it is only recently since the development of molecular DIVA tools that differentiate vaccinal from wild type strains [10], [11] that this has come to light. Our study has also identified a field strain (AB15) from an animal that was reported as not being vaccinated but in contact with animals that had been vaccinated as being almost identical to strain 1B. This is first experimental evidence suggesting the transmission of the vaccinal strain from animal to animal. However, this interpretation is dependent on the accuracy of the vaccine records for the farm in question and cannot discount possible mistakes in record keeping. This finding is supported by the additional SNPs found in strains 11-669_5380/2 and 10DC0084, suggesting that the vaccine strain may even be circulating in flocks and mutating over time. However, further evidence is required to confirm this possibility.

If the vaccine strain can cause disease it does raise questions as to how and why this is seen in some animals and not others. The live vaccine strain is thought to elicit protection without causing disease due to attenuation resulting in absence of growth of the pathogen within the animal at 39.5 °C (the body temperature of sheep). However, our study has failed to identify the genetic basis of such a temperature-sensitive phenotype, suggesting that the pathogen does have the capacity to multiply within the animal and infect the placenta causing disease. However, an important observation was noted in a study we published in 2013 [22] that may explain why we do not observe disease in all animals. In this study, we observed that inoculation of ewes prior to pregnancy with low (5 × 10^3^ IFU) and medium (5 × 10^5^ IFU) doses of *C. abortus* resulted in infection and abortion typical of what is observed with the disease in the field, while inoculation with a high dose (5 × 10^7^ IFU) stimulated a protective immune response [22]. As the commercial vaccines are administered at a dosage ranging 10^5^–10^6.9^ IFU *C. abortus*, it is possible that, depending on the exact titre of a particular batch and whether or not the vaccines are administered in accordance with manufacturers’ instructions, the dose may be low enough for abortions to occur or high enough to effectively protect the flock. Clearly, this would have important implications for consistency in manufacturing and for those administering the vaccines to carefully follow the manufacturers’ instructions. Therefore, taking all these results and points into account we suggest that protection or otherwise of the 1B vaccine is unlikely to be due to any chemically induced SNPs and more likely dependent on the exact titre of a particular batch of vaccine and the care taken in following manufacturers’ instruction during vaccine reconstitution and the inoculation of the animals prior to pregnancy.

## Disclaimers

The views expressed in the submitted article are the opinions of the authors of this publication and do not represent an official position of any of the institutions in which they work or of any of the funders.

## Source(s) of support

Wellcome (Grant No. 098051), the Biotechnology and Biological Sciences Research Council (BBSRC) (Grant No. BB/E018939/1) and the Scottish Government Rural and Environment Science and Analytical Services division (RESAS).

## Appendix A Supplementary material

Supplementary data 1 **Fig. S1**. Mapping of the AB7 and 1B SNPs identified in Burall et al. (2009) to the AB7 assembly in this study. Mapped reads are shown in the top panels, with non-matching bases (sequencing errors and SNPs) coloured red. A representative screenshot of the AB7 mapped reads (133x mean coverage) and the 1B-Cevac mapped reads (299x mean coverage) is shown in each panel.

Supplementary data 2 **Table S1.** SNPs and indels in *C. abortus* strain AB7 relative to strain S26/3. **Table S2.** Non-synonymous mutations and indels affecting protein coding sequences in *C. abortus* strain AB7 relative to strain S26/3. **Table S3.** Pseudogene content in *C. abortus* strain AB7 relative to strain S26/3.
