# Supplementary material for: Genomic evidence that the live Chlamydia abortus vaccine strain 1B is not attenuated and has the potential to cause disease
Source: Vaccine. 2018 Jun 14;36(25):3593–8. doi: 10.1016/j.vaccine.2018.05.042 (PMC6005232; doi:10.1016/j.vaccine.2018.05.042)
Supplement: Supplementary data 1 — Fig. S1. Mapping of the AB7 and 1B SNPs identified in Burall et al. (2009) to the AB7 assembly in this study. Mapped reads are shown in the top panels, with non-matching bases (sequencing errors and SNPs) coloured red. A representative screenshot of the AB7 mapped reads (133x mean coverage) and the 1B-Cevac mapped reads (299x mean coverage) is shown in each panel. [file mmc1.pptx]

## Slide 1
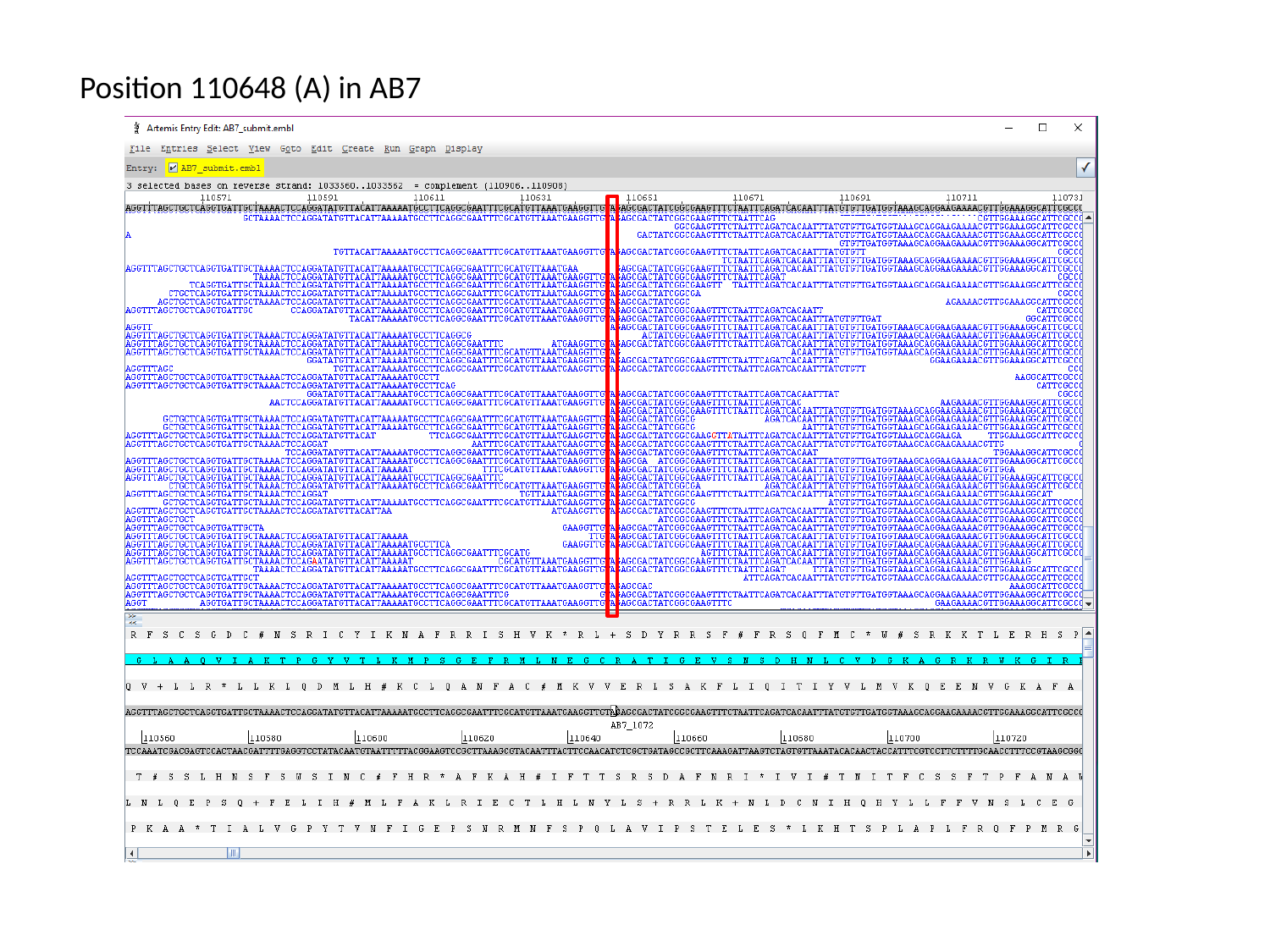

Position 110648 (A) in AB7

## Slide 2
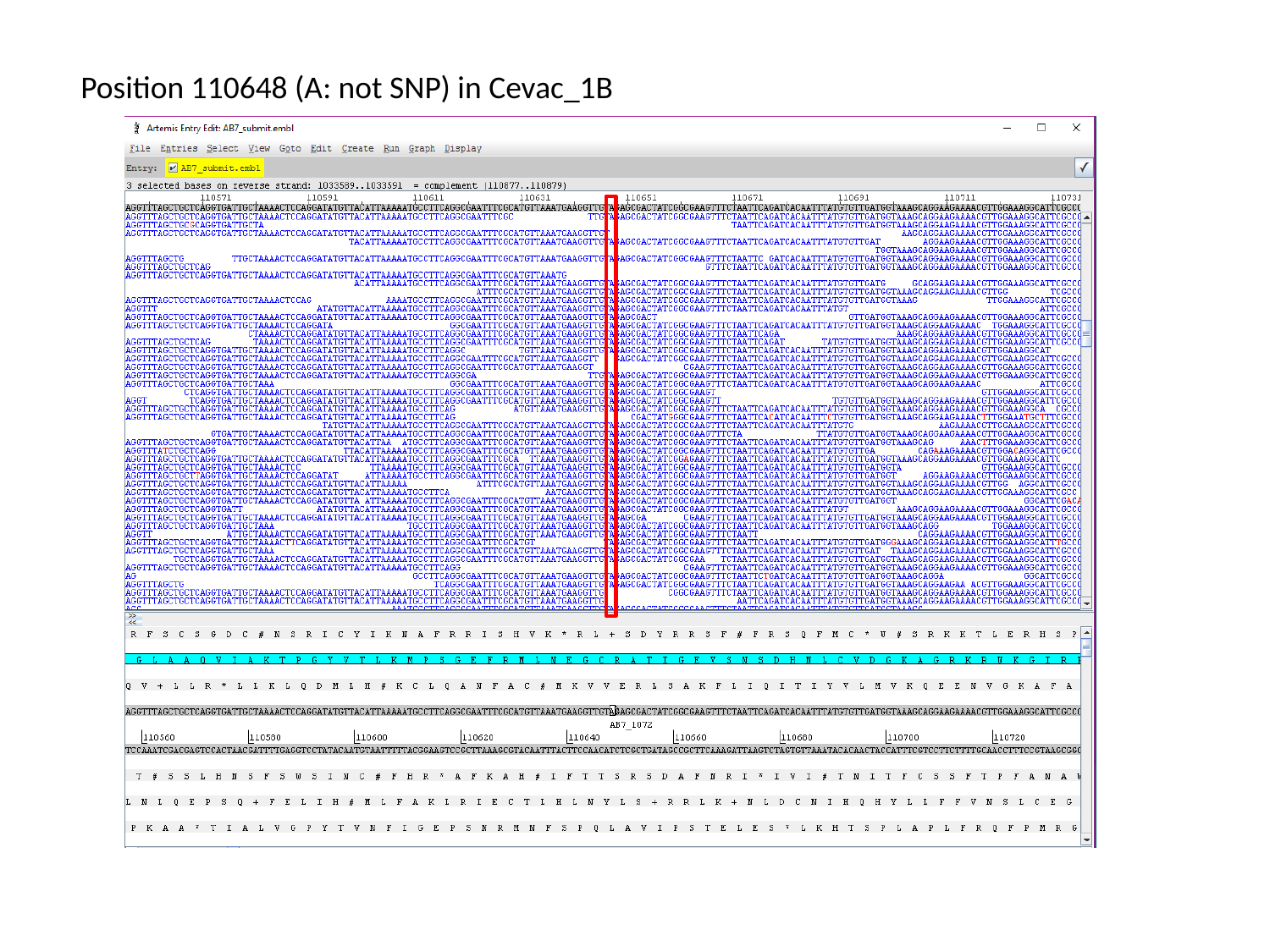

Position 110648 (A: not SNP) in Cevac_1B

## Slide 3
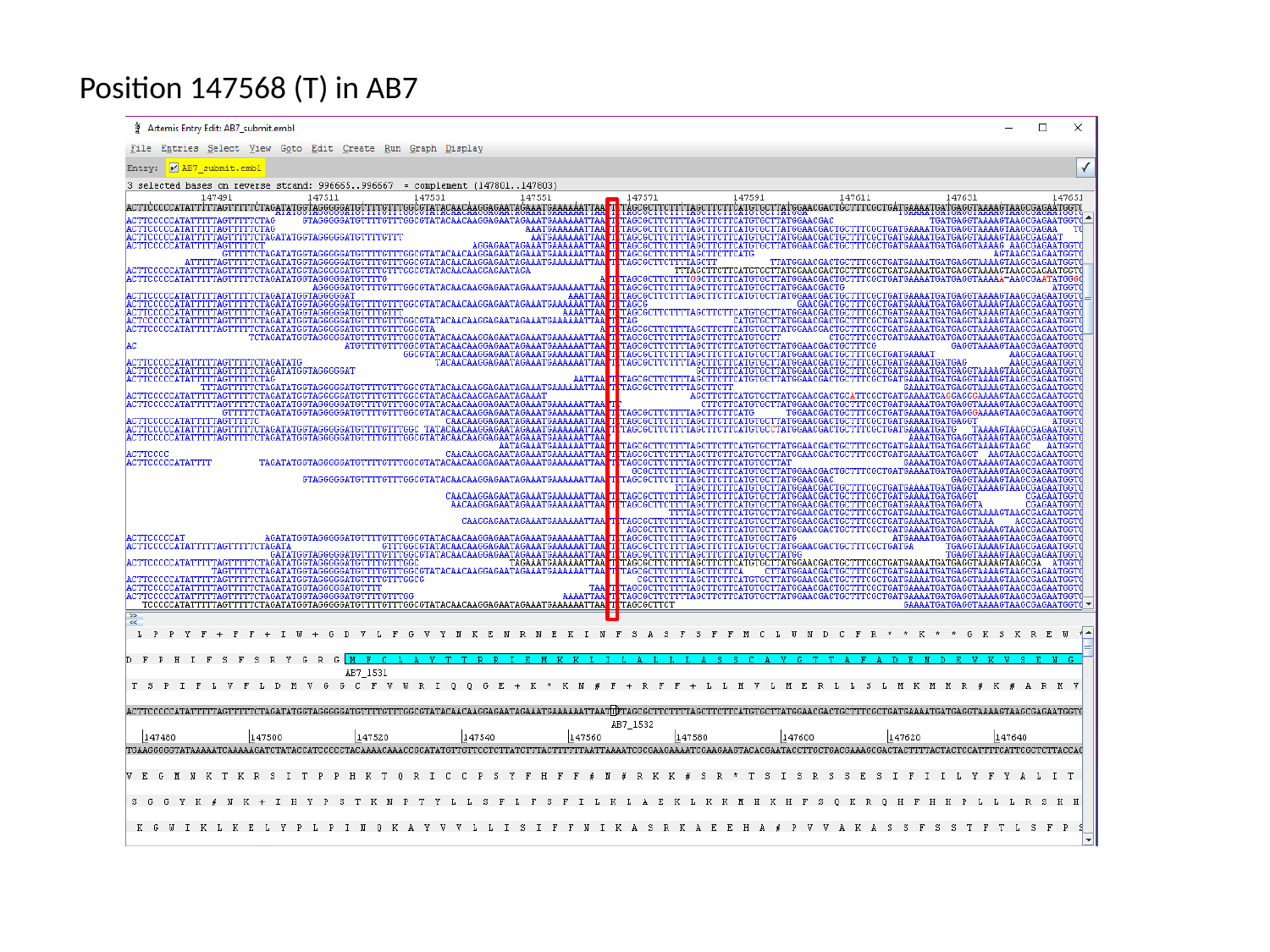

Position 147568 (T) in AB7

## Slide 4
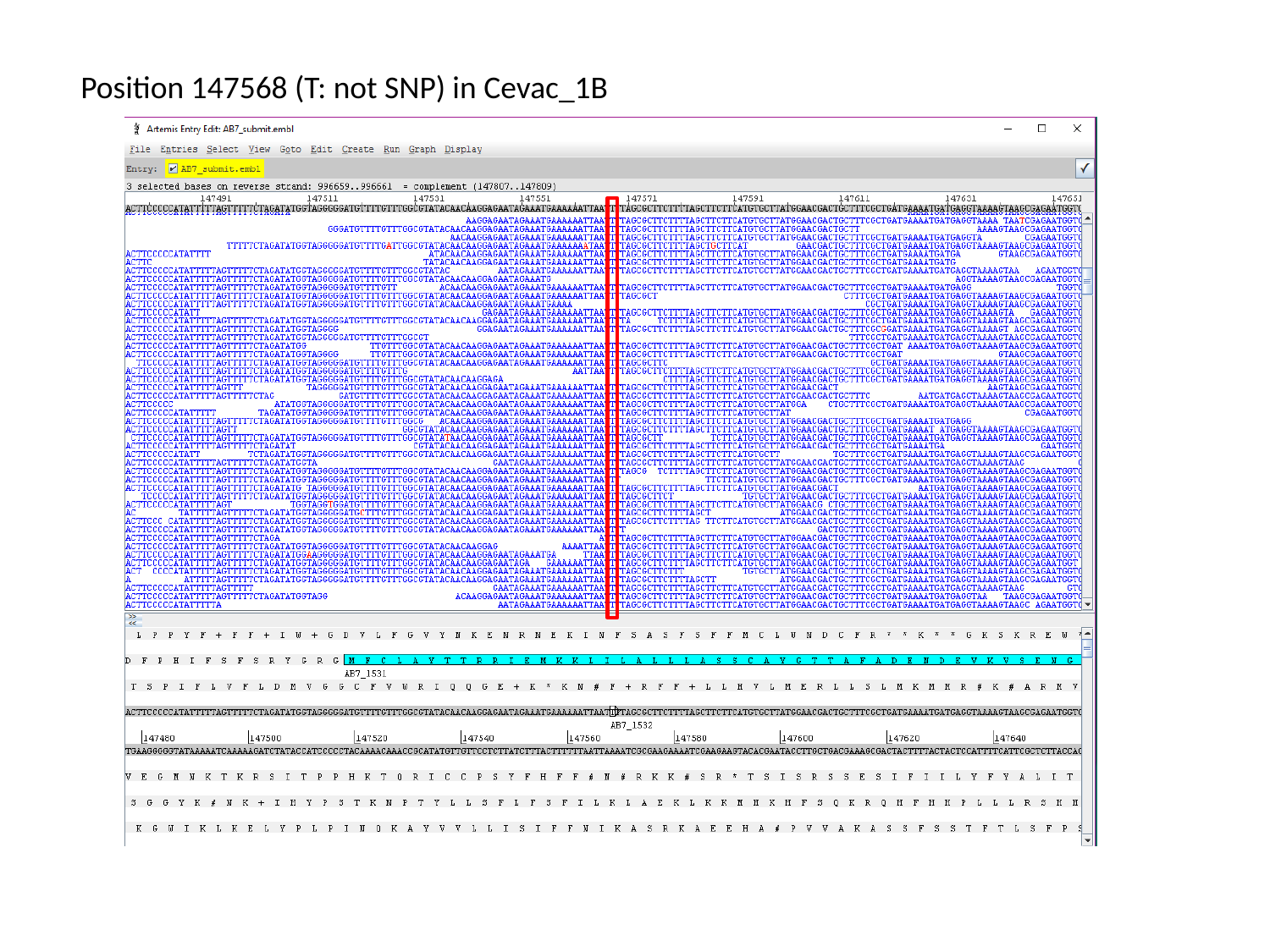

Position 147568 (T: not SNP) in Cevac_1B

## Slide 5
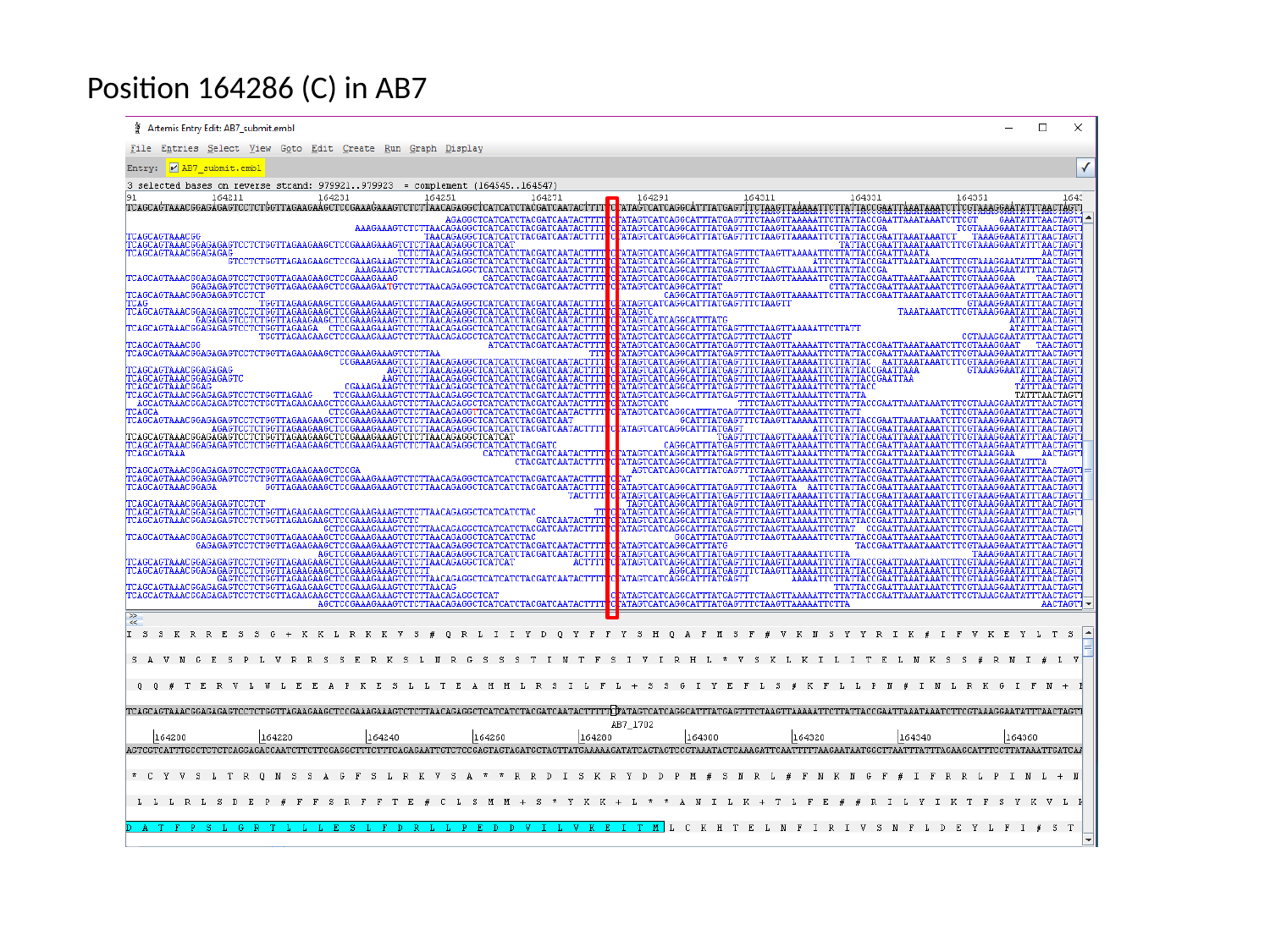

Position 164286 (C) in AB7

## Slide 6
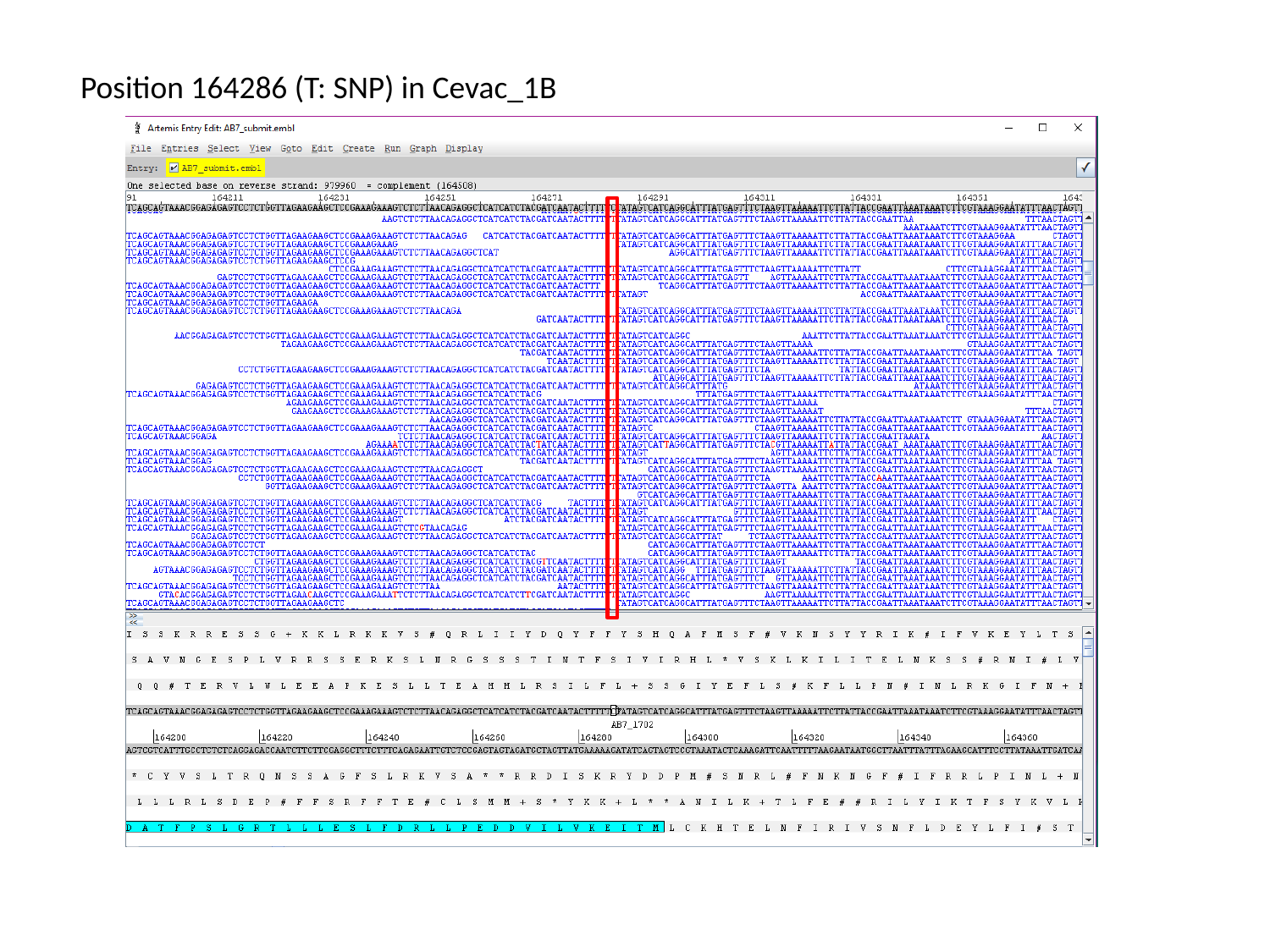

Position 164286 (T: SNP) in Cevac_1B

## Slide 7
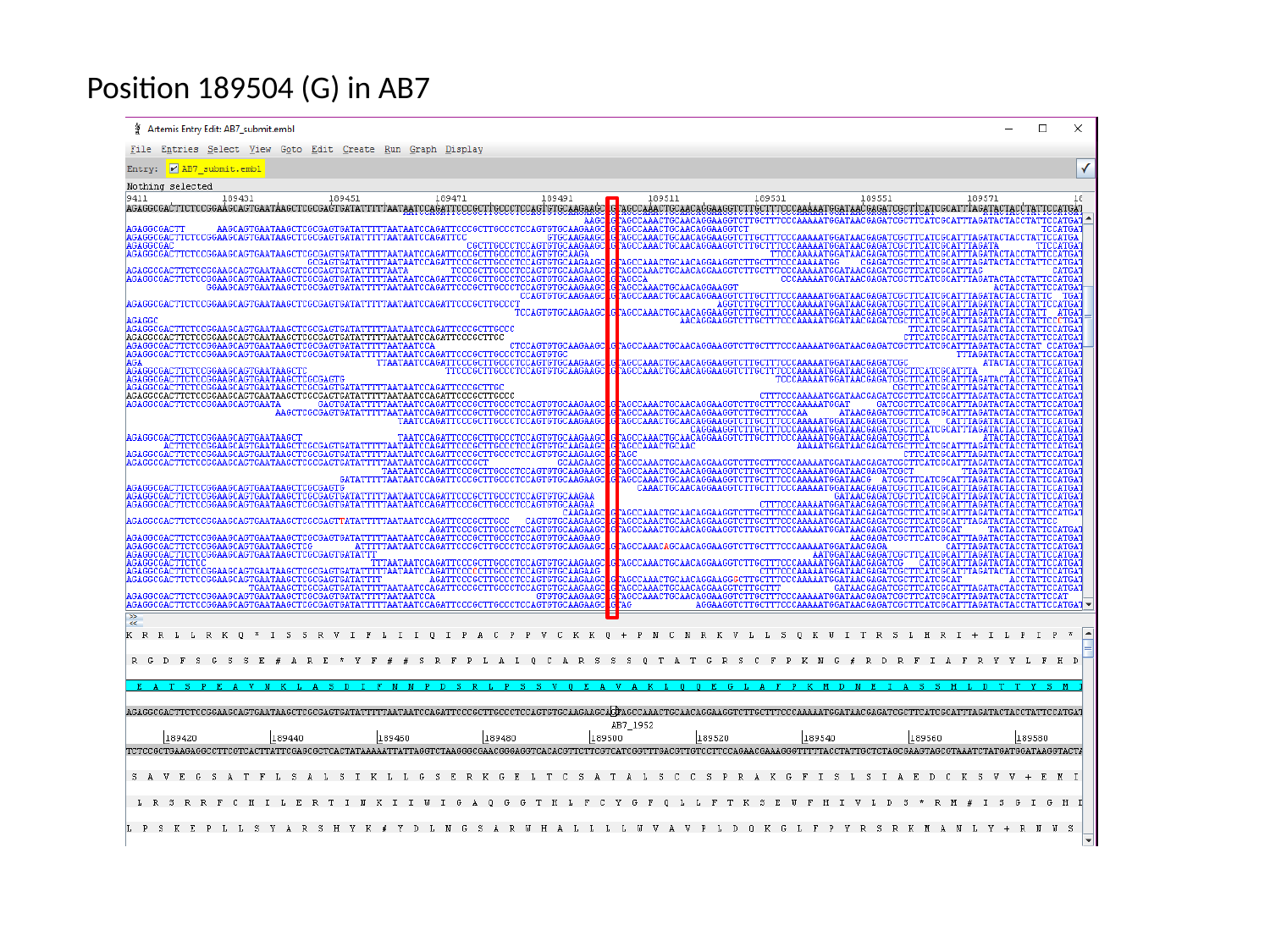

Position 189504 (G) in AB7

## Slide 8
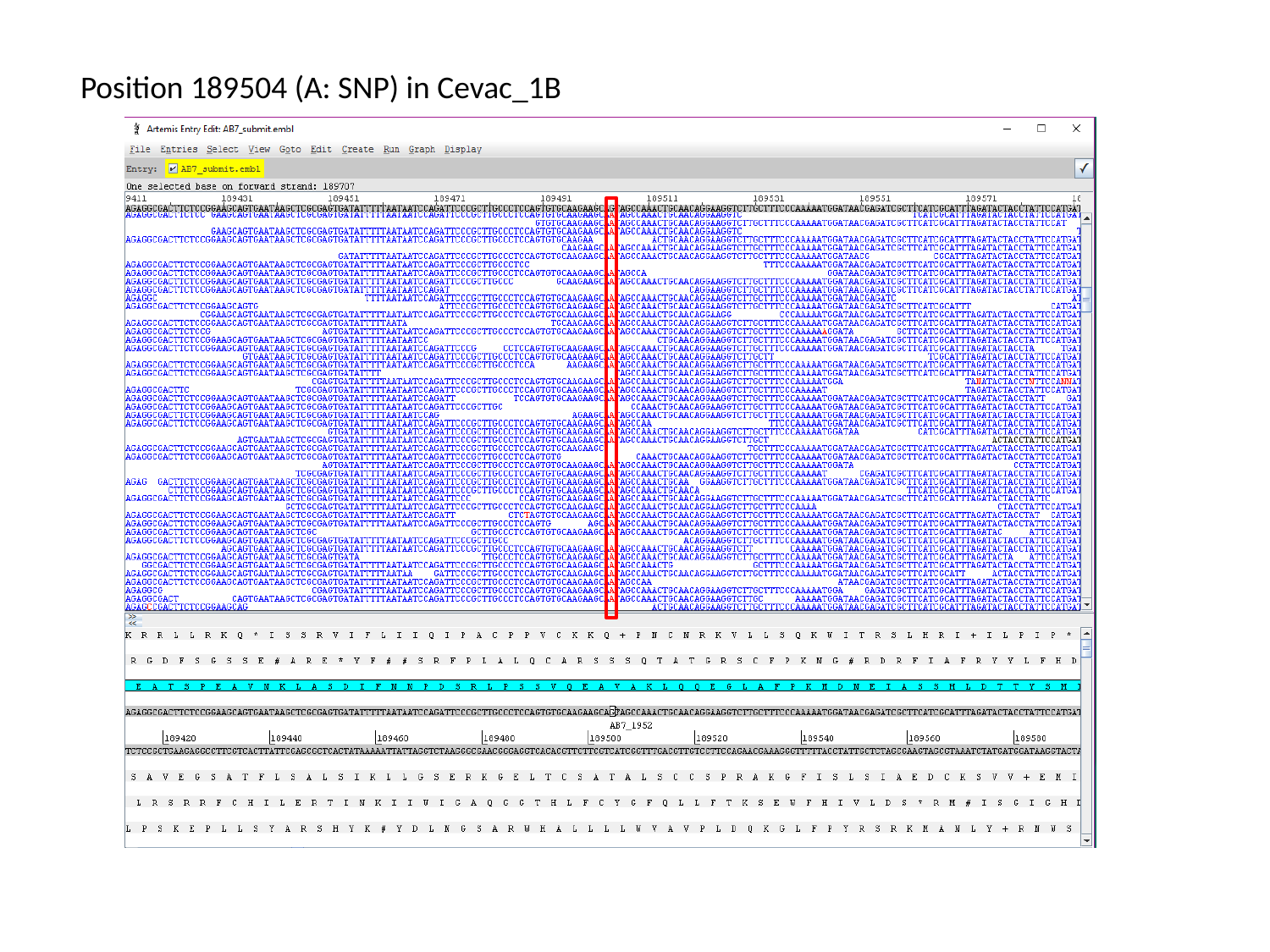

Position 189504 (A: SNP) in Cevac_1B

## Slide 9
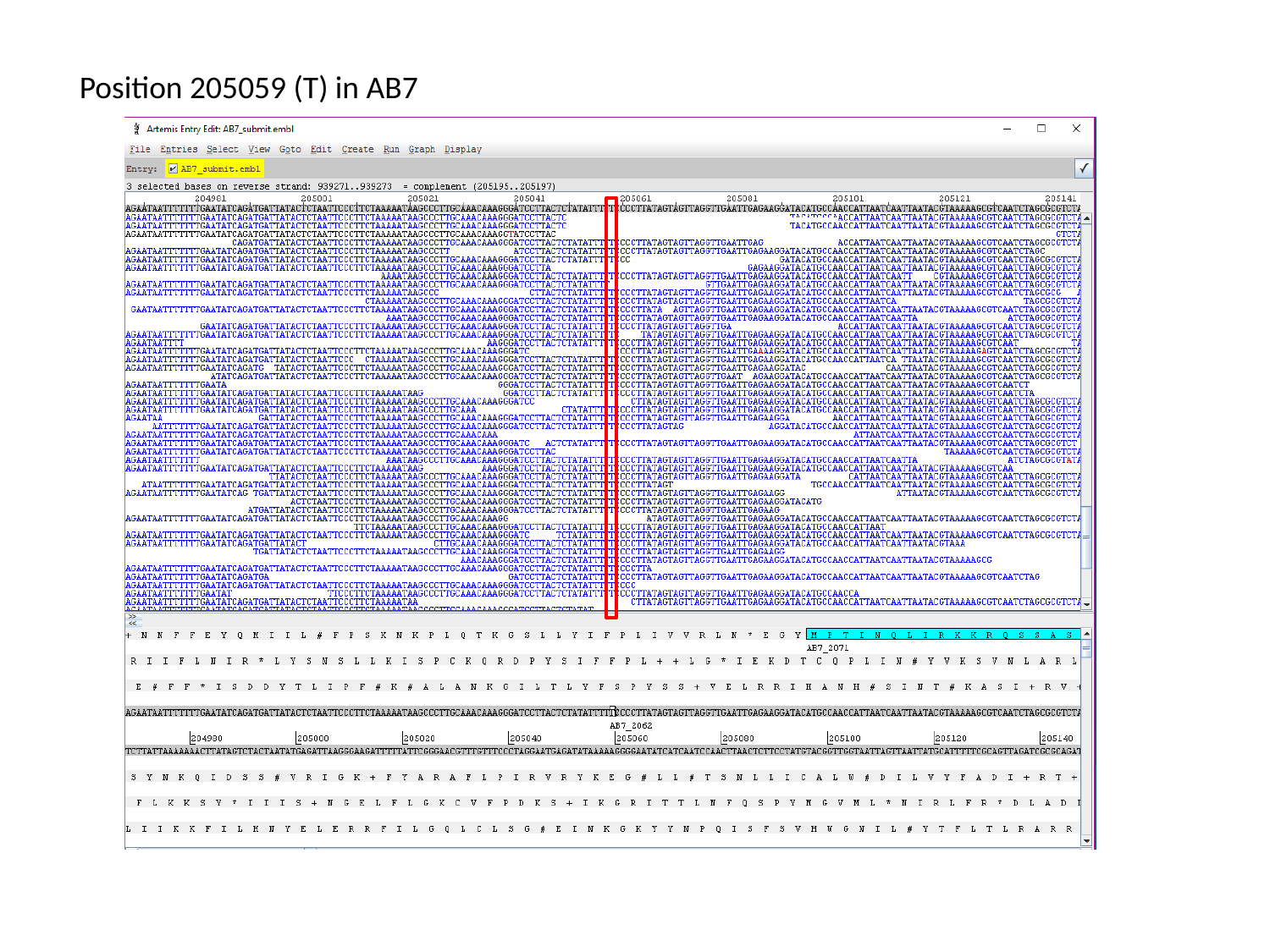

Position 205059 (T) in AB7

## Slide 10
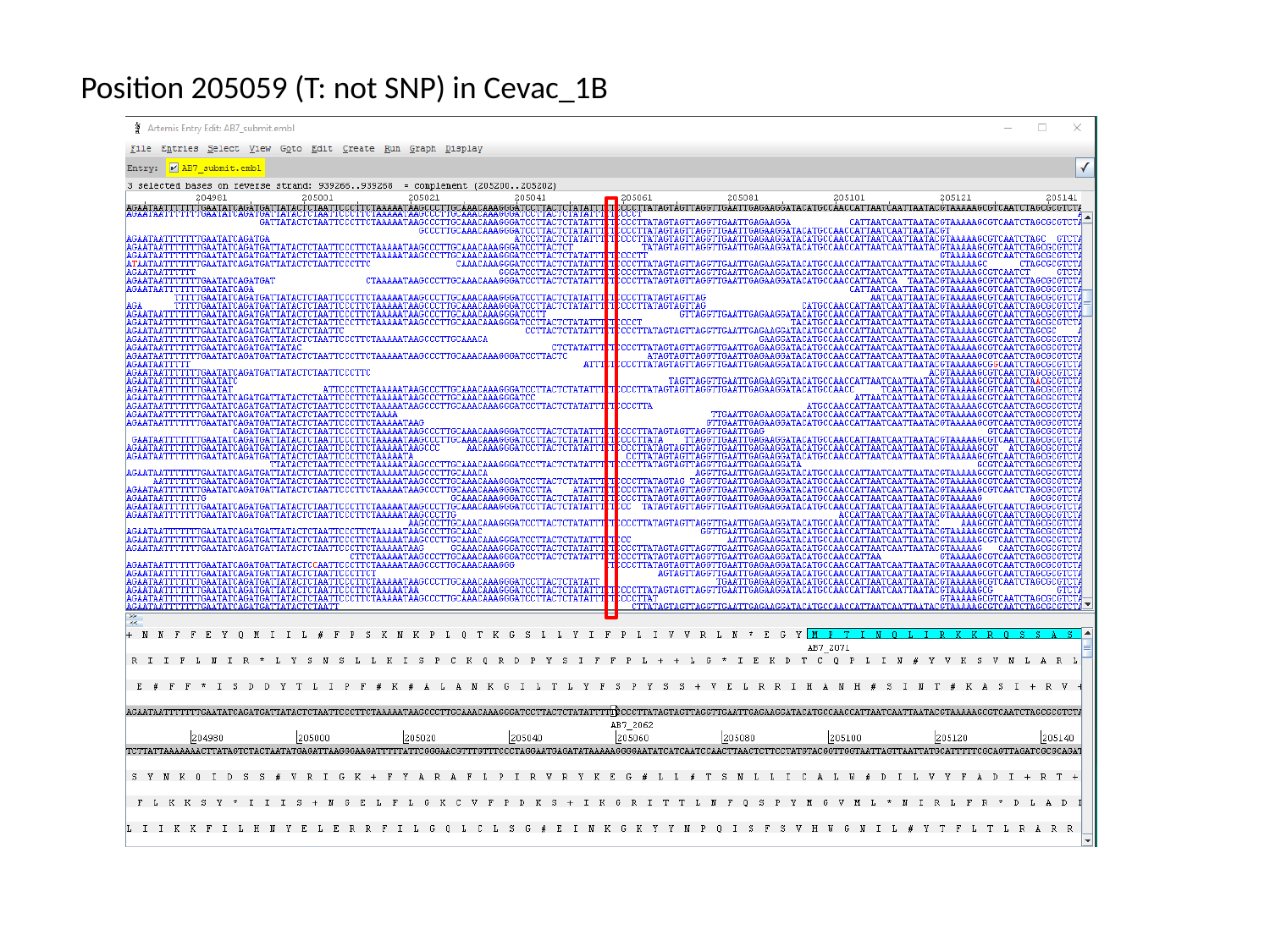

Position 205059 (T: not SNP) in Cevac_1B

## Slide 11
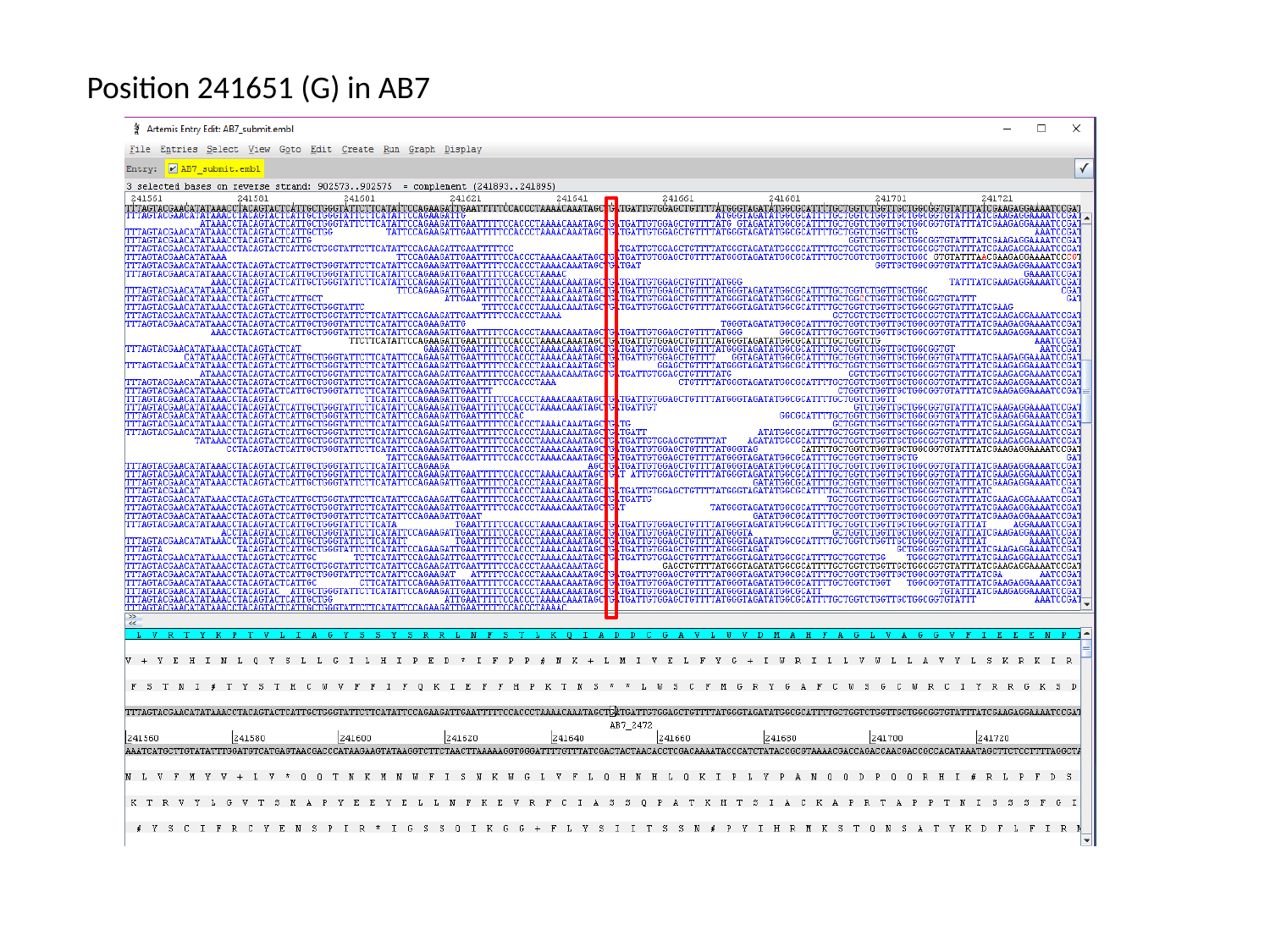

Position 241651 (G) in AB7

## Slide 12
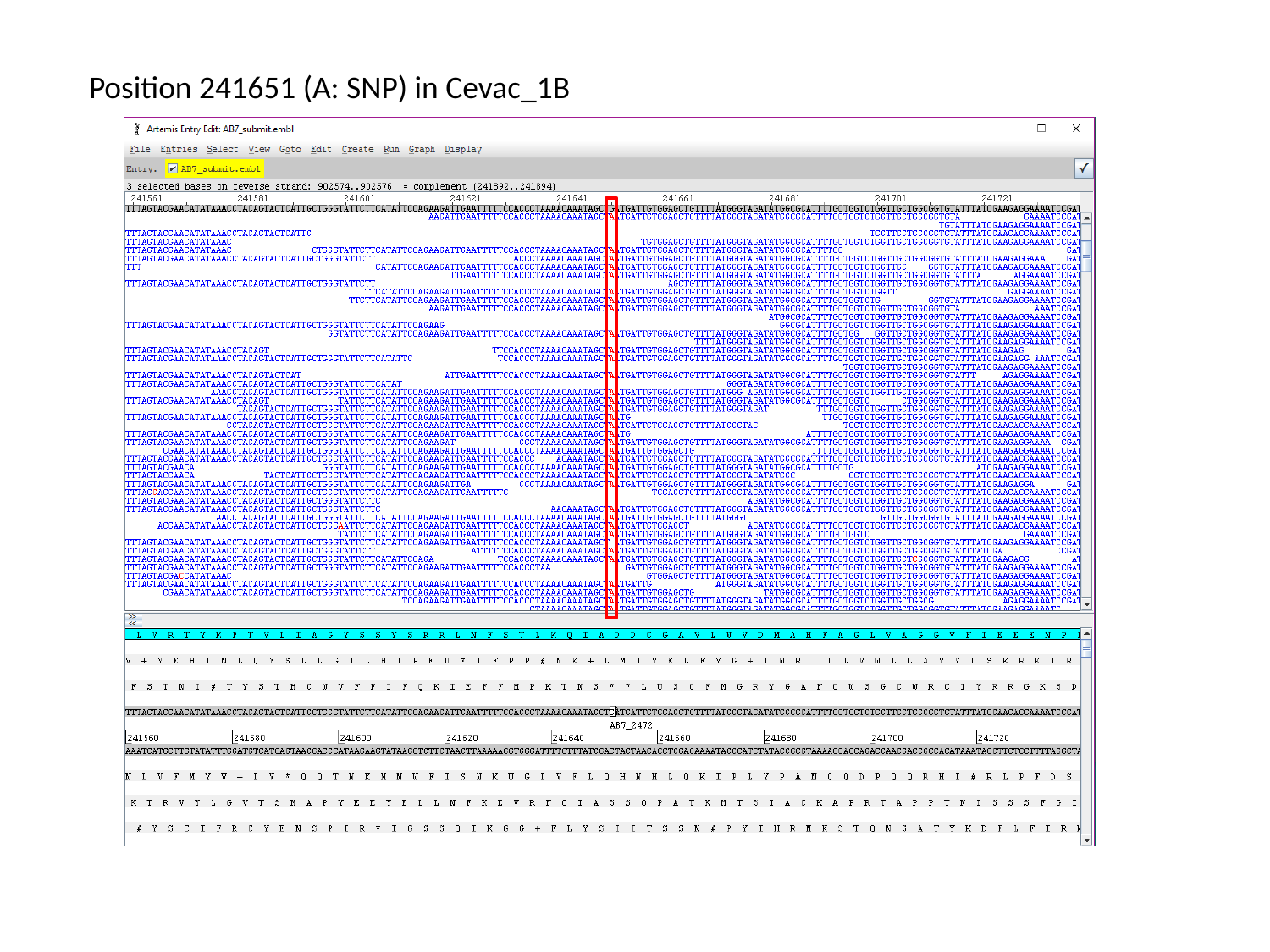

Position 241651 (A: SNP) in Cevac_1B

## Slide 13
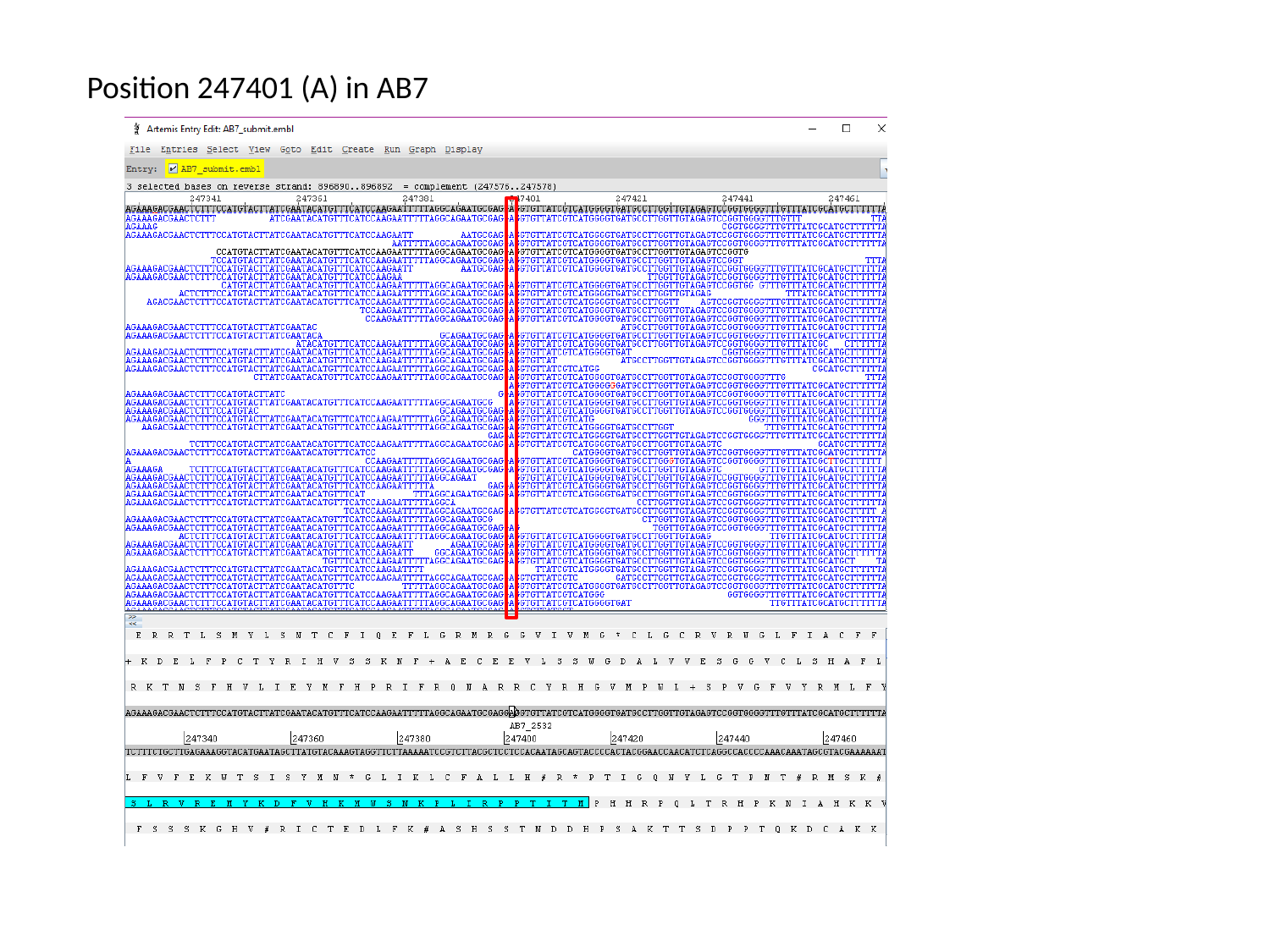

Position 247401 (A) in AB7

## Slide 14
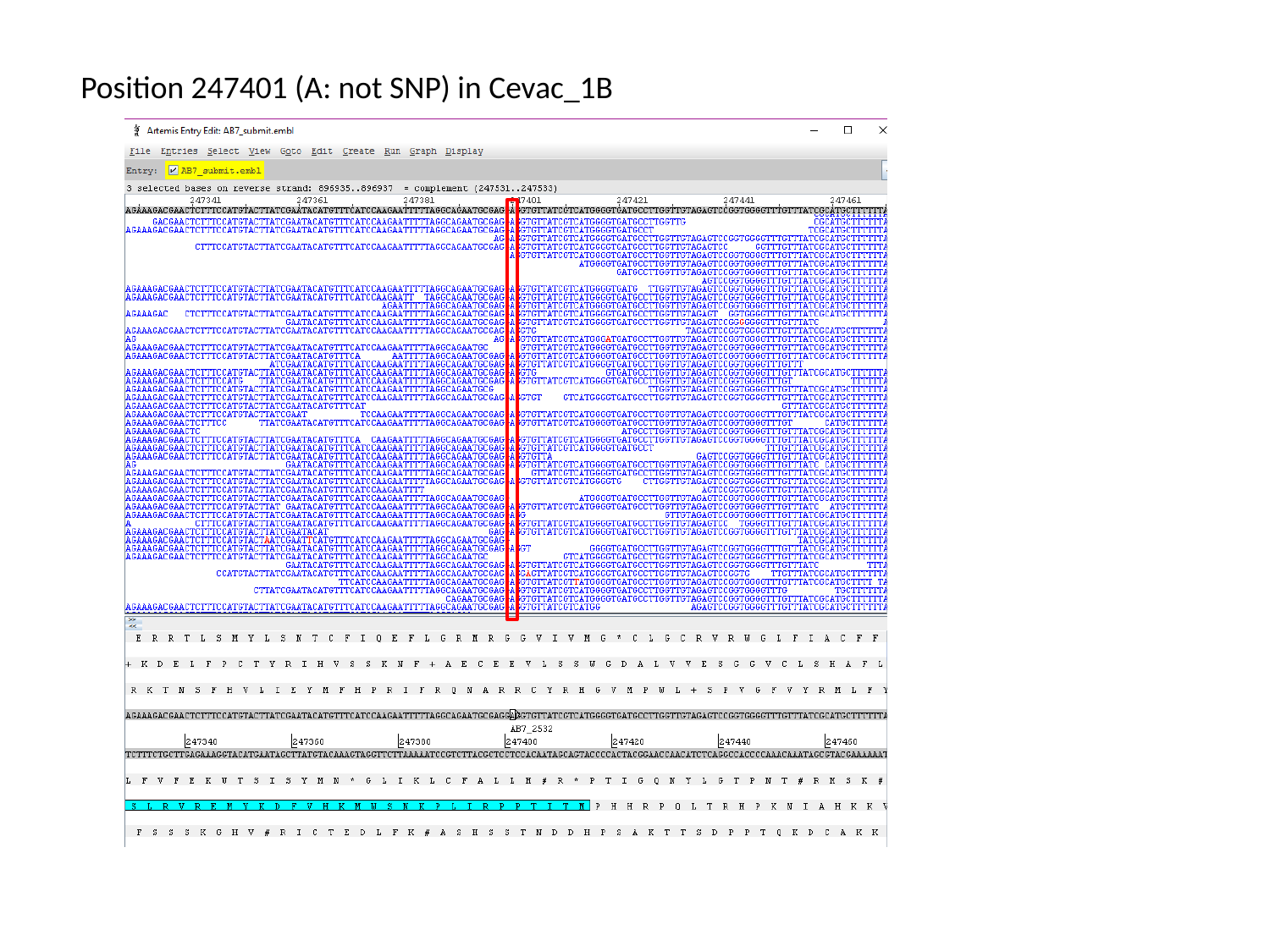

Position 247401 (A: not SNP) in Cevac_1B

## Slide 15
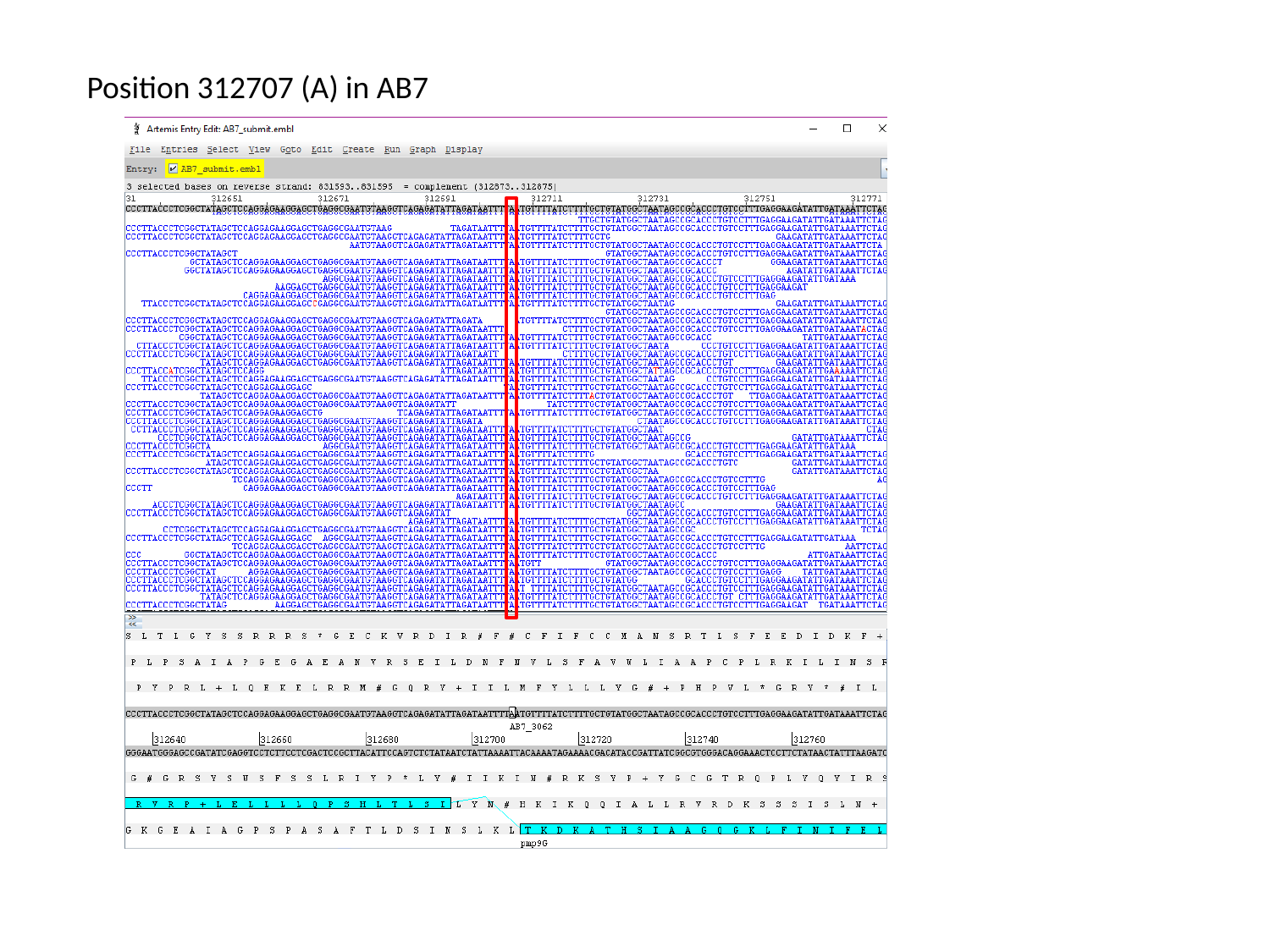

Position 312707 (A) in AB7

## Slide 16
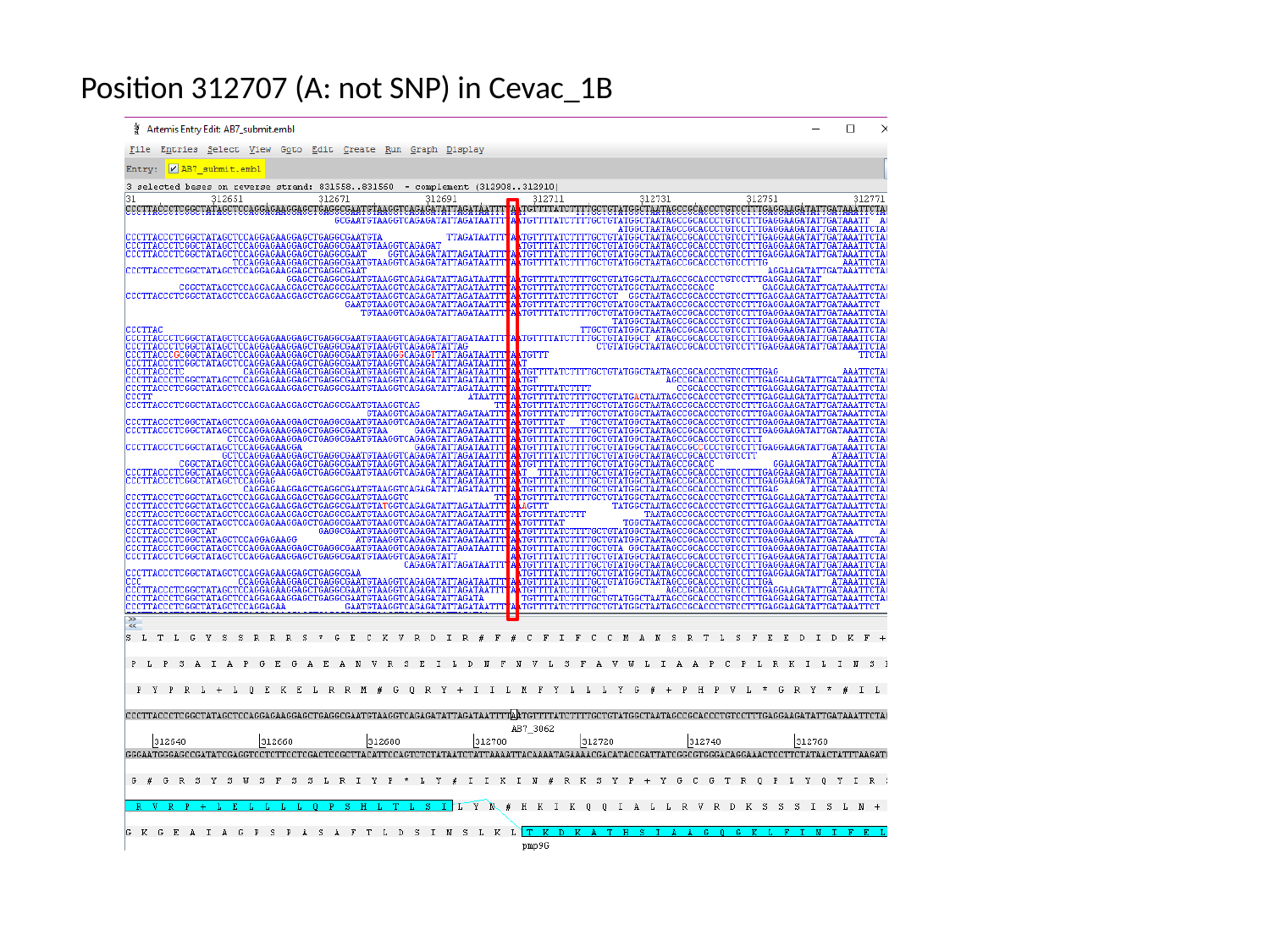

Position 312707 (A: not SNP) in Cevac_1B

## Slide 17
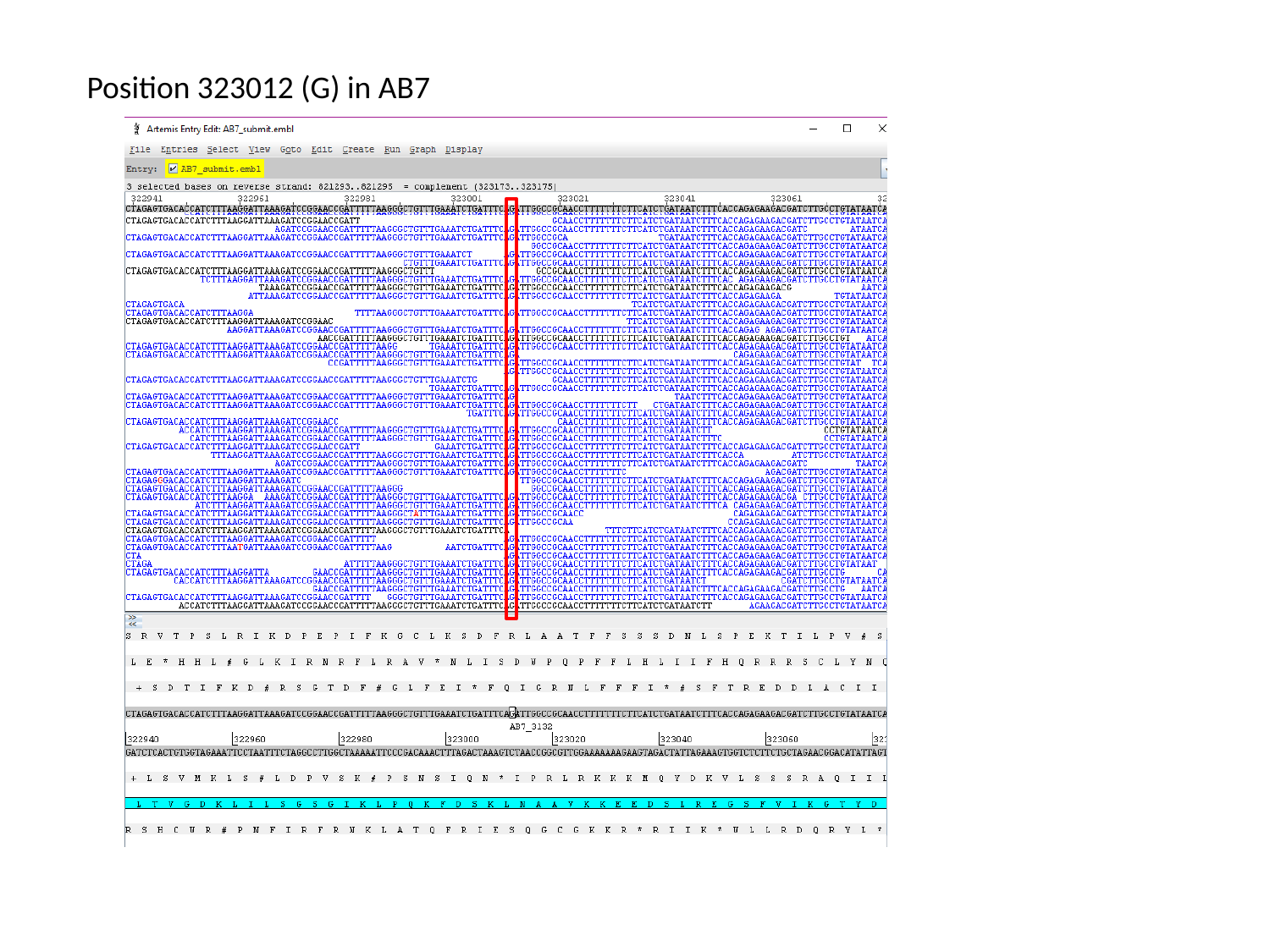

Position 323012 (G) in AB7

## Slide 18
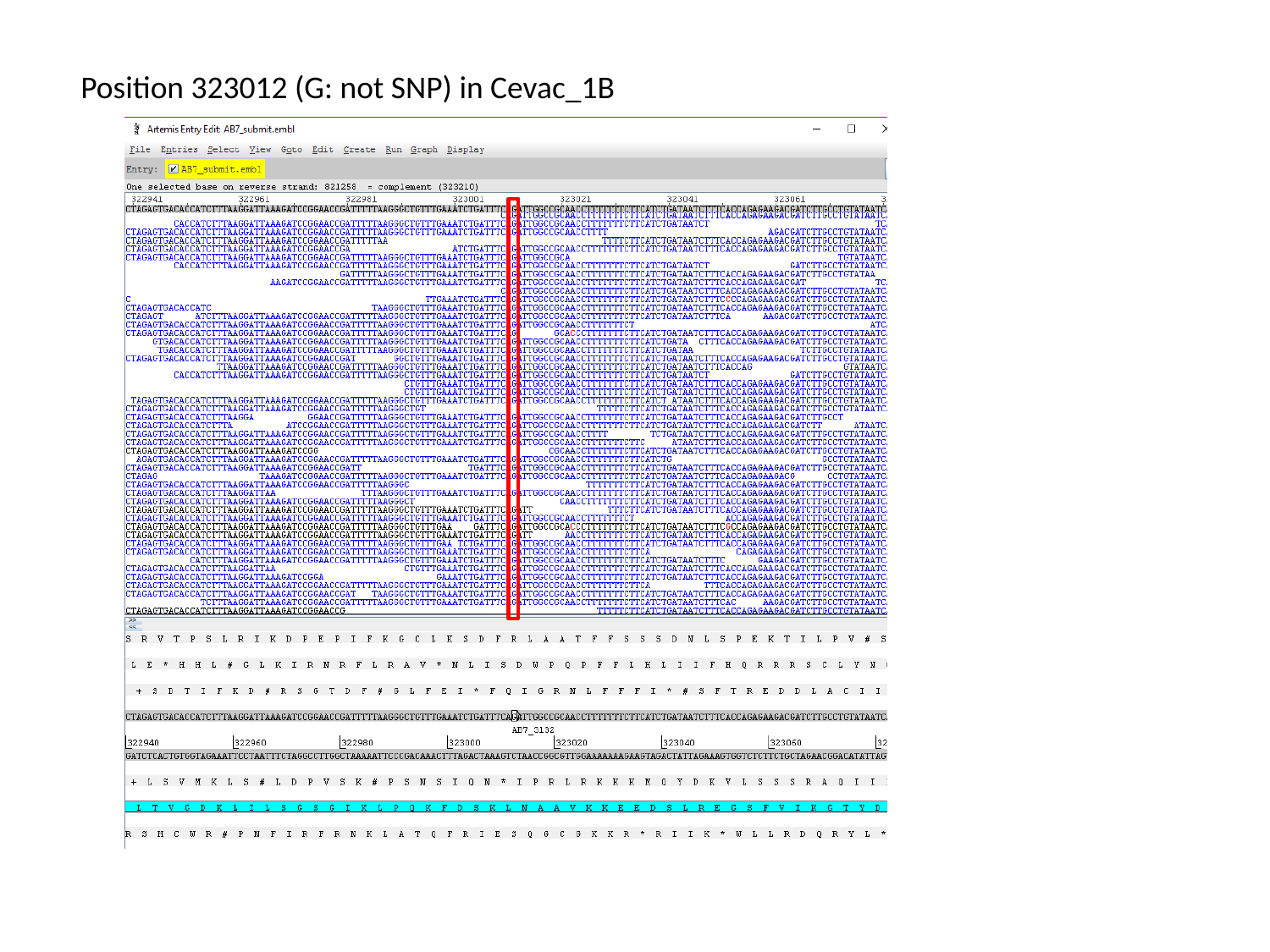

Position 323012 (G: not SNP) in Cevac_1B

## Slide 19
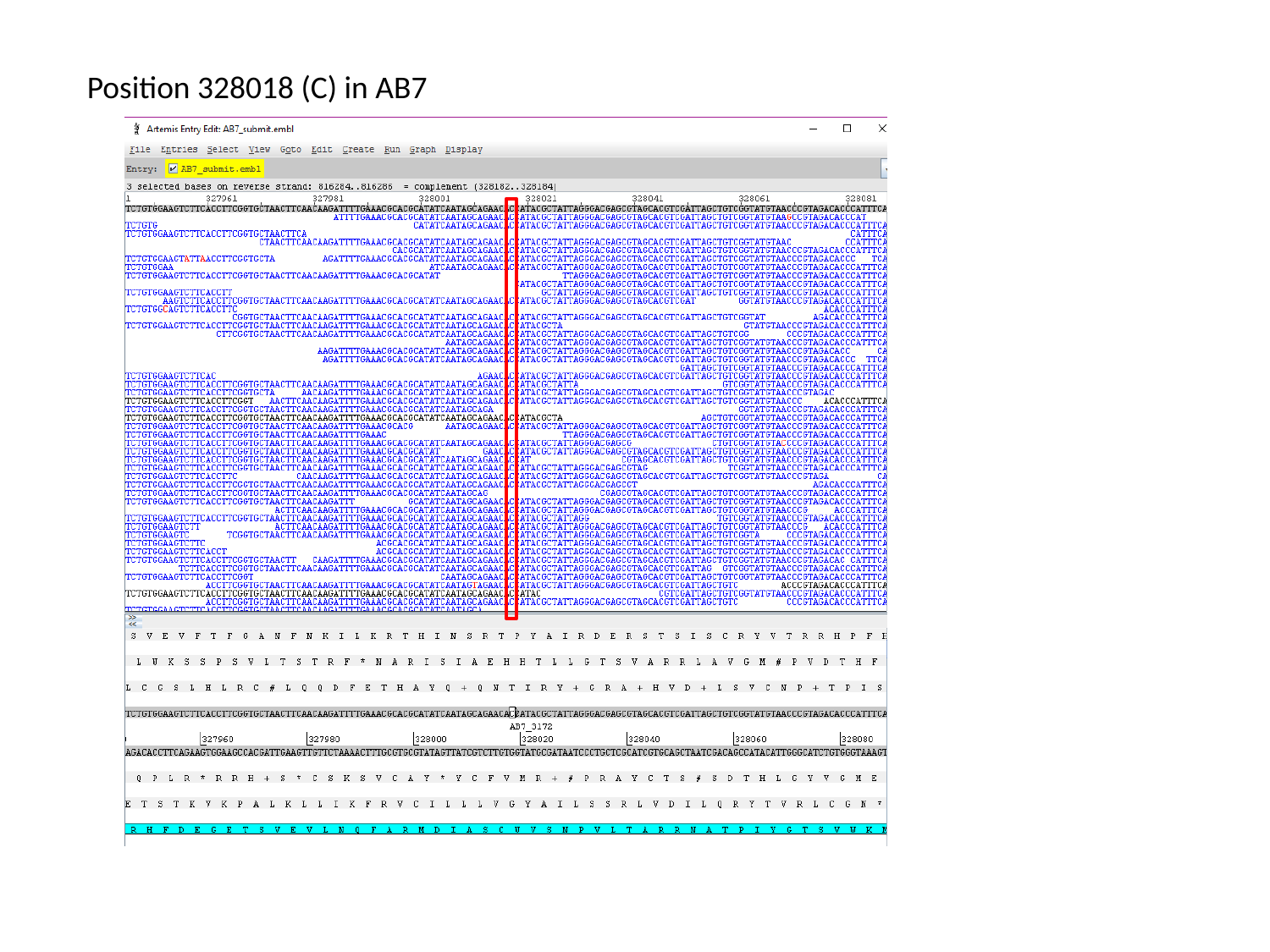

Position 328018 (C) in AB7

## Slide 20
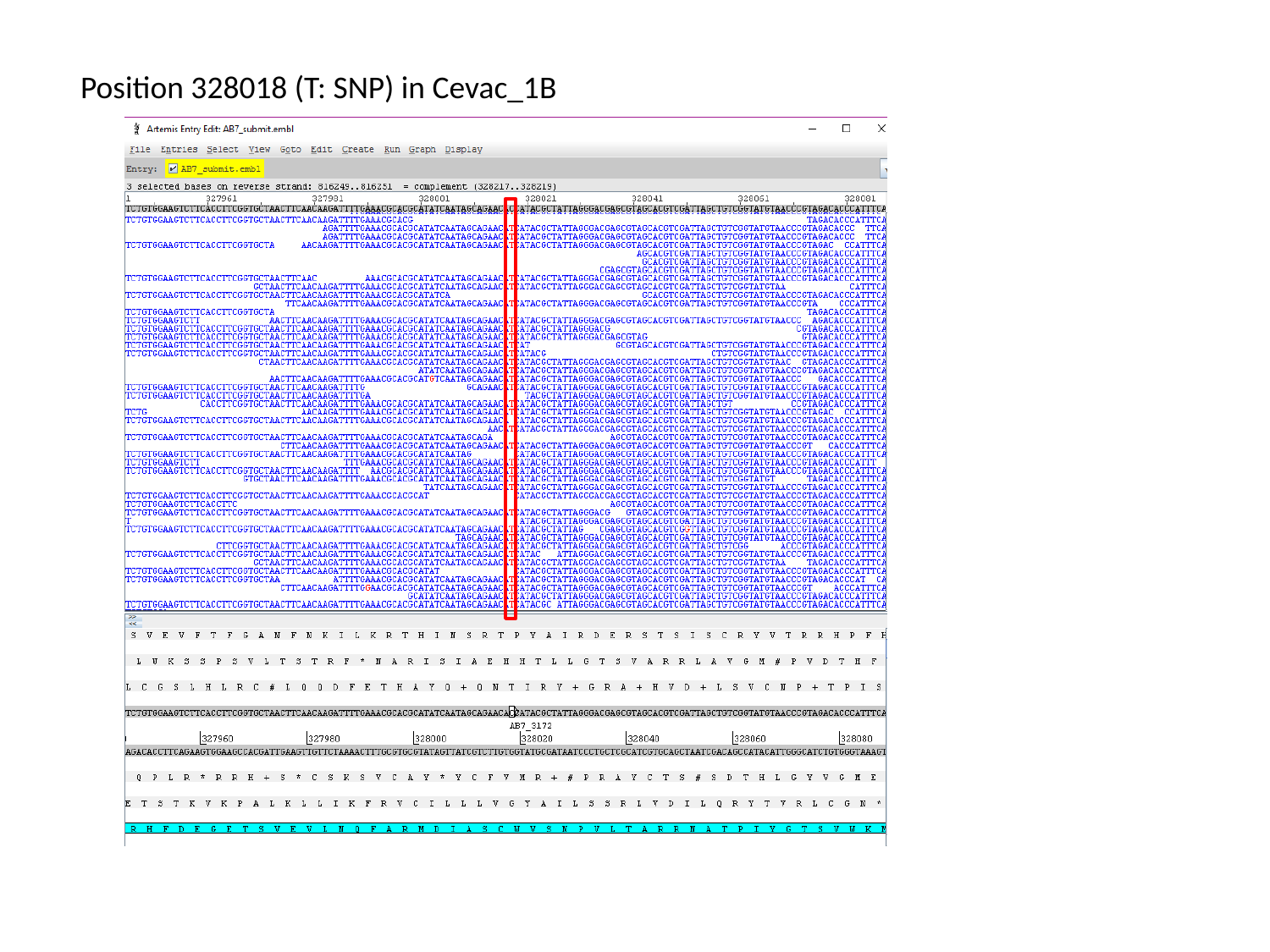

Position 328018 (T: SNP) in Cevac_1B

## Slide 21
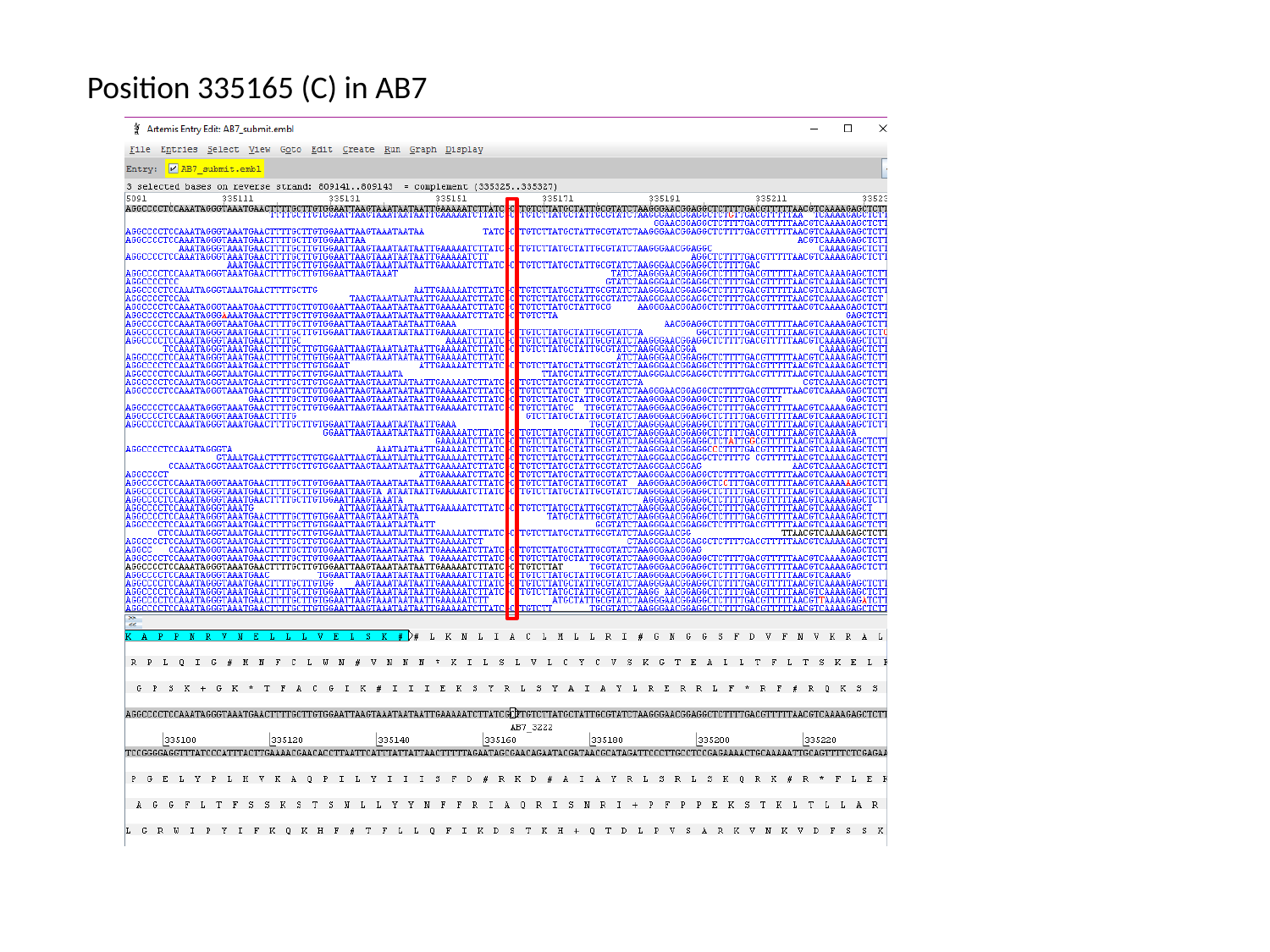

Position 335165 (C) in AB7

## Slide 22
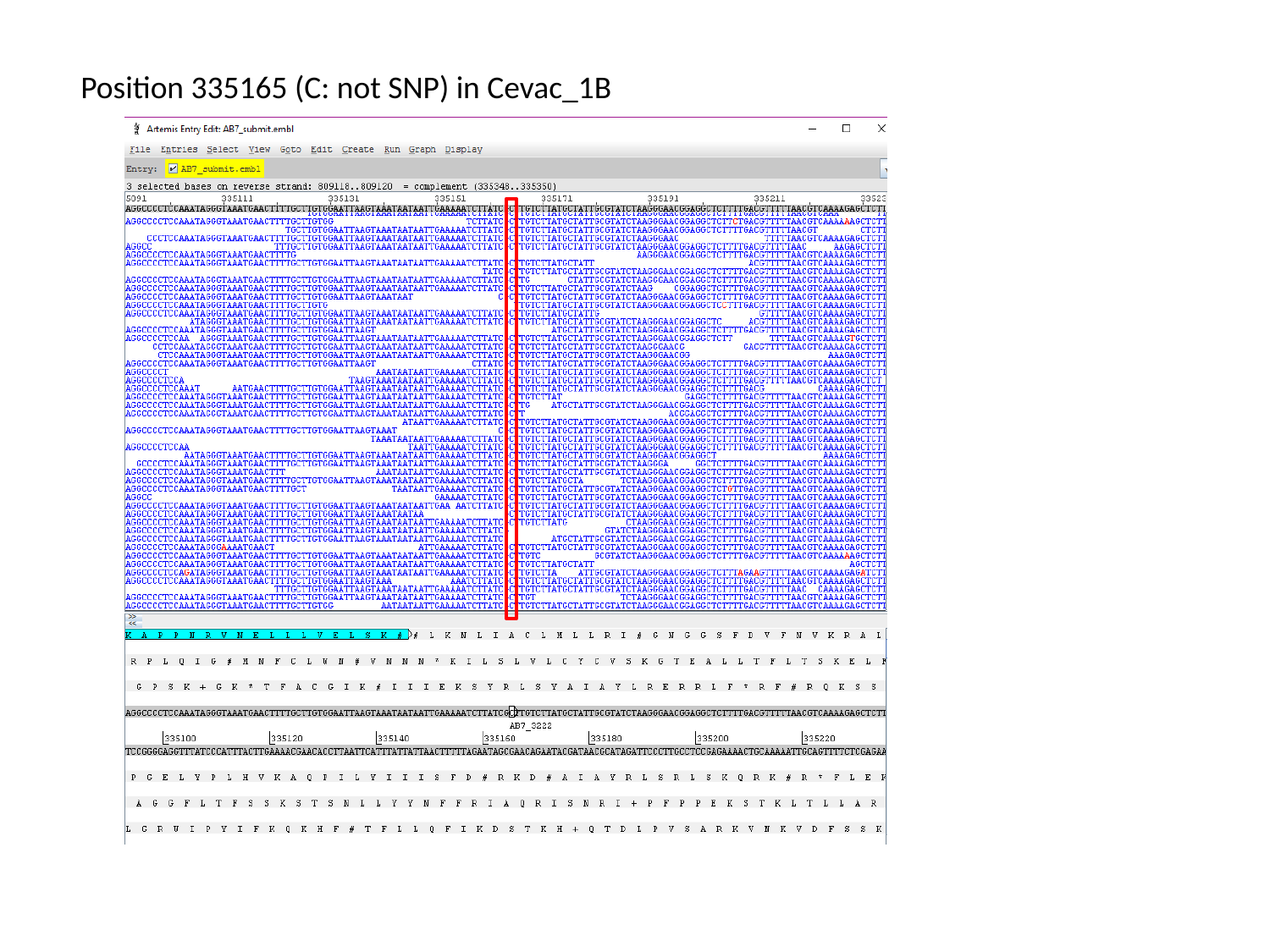

Position 335165 (C: not SNP) in Cevac_1B

## Slide 23
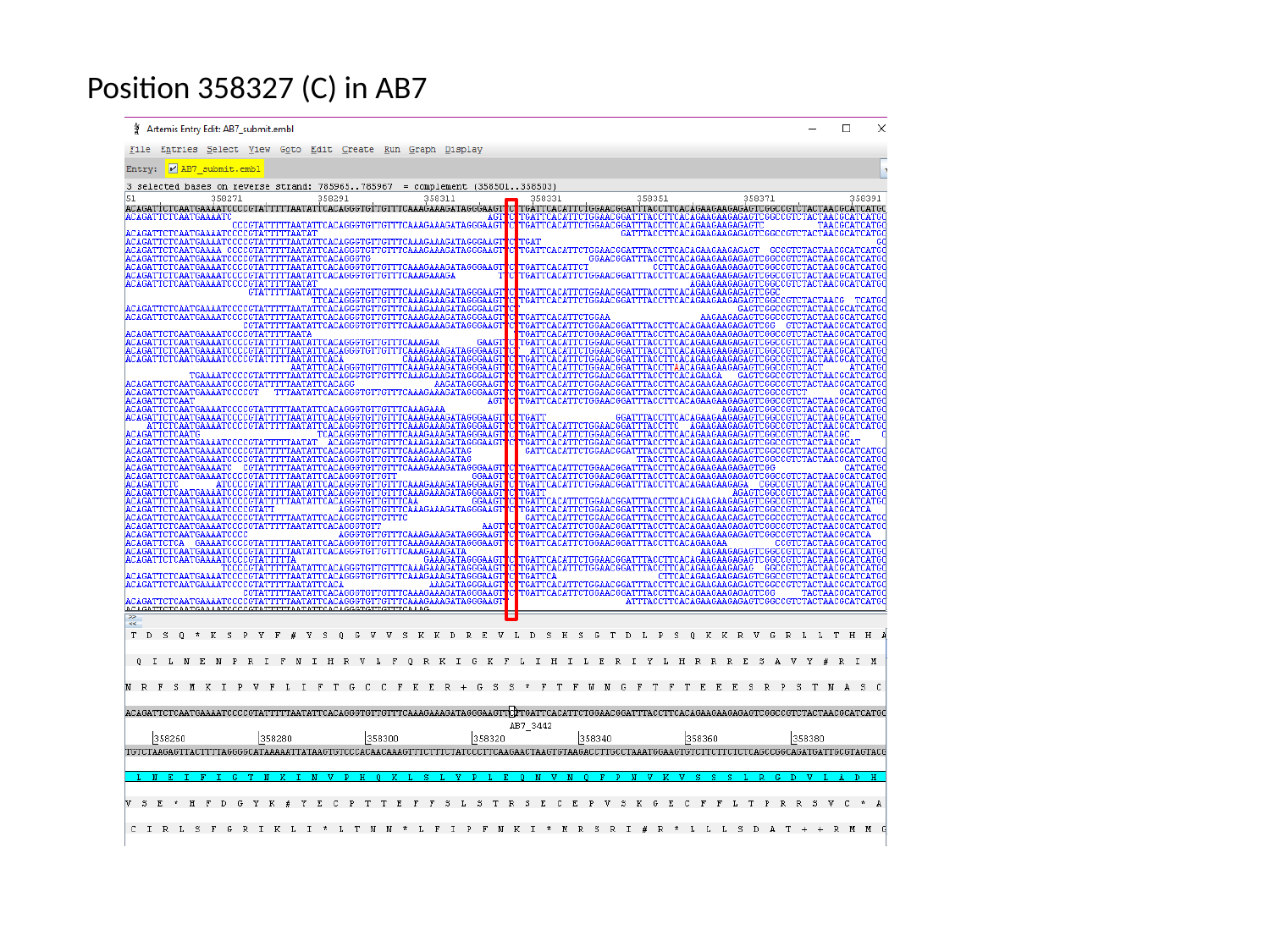

Position 358327 (C) in AB7

## Slide 24
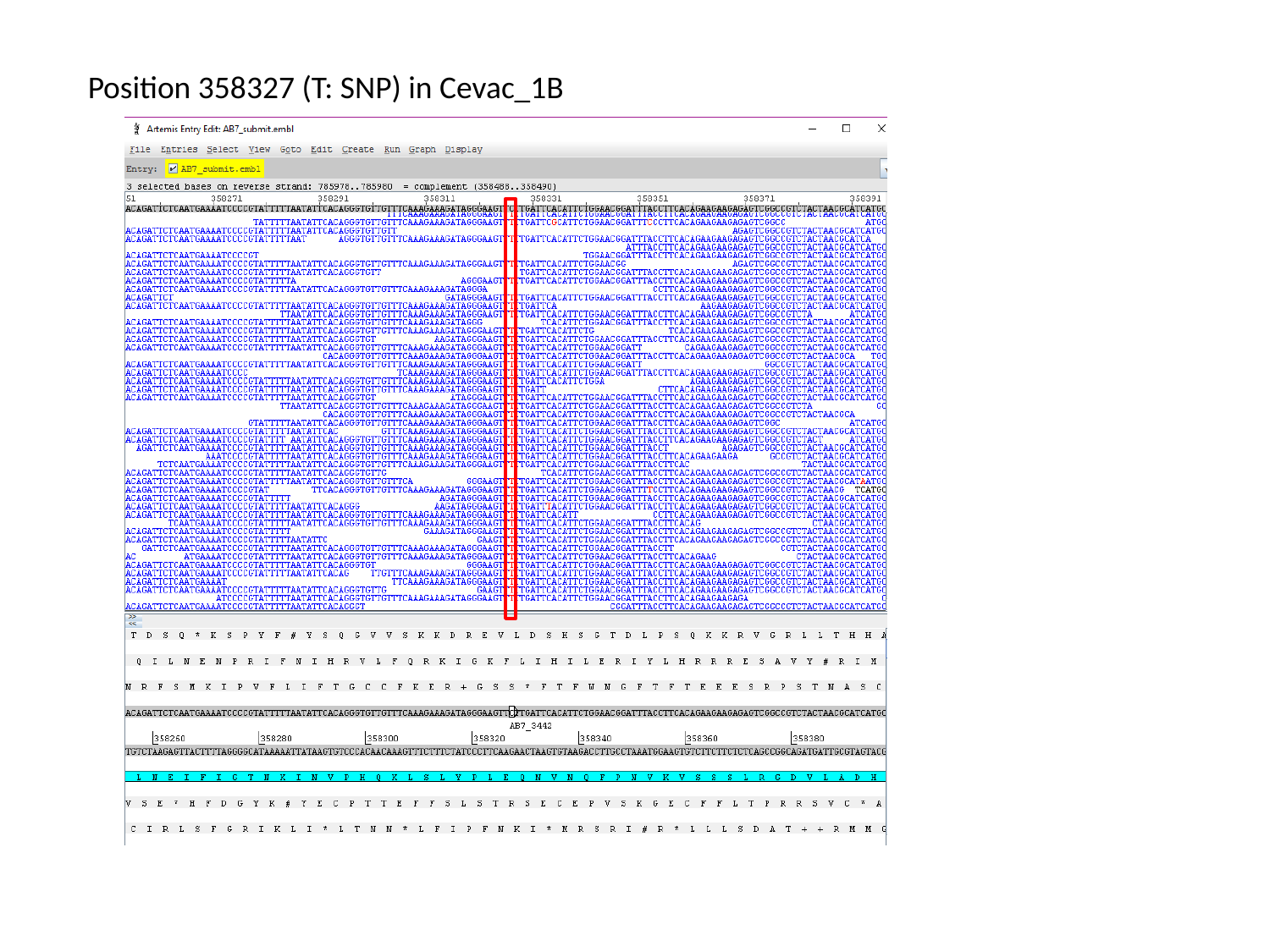

Position 358327 (T: SNP) in Cevac_1B

## Slide 25
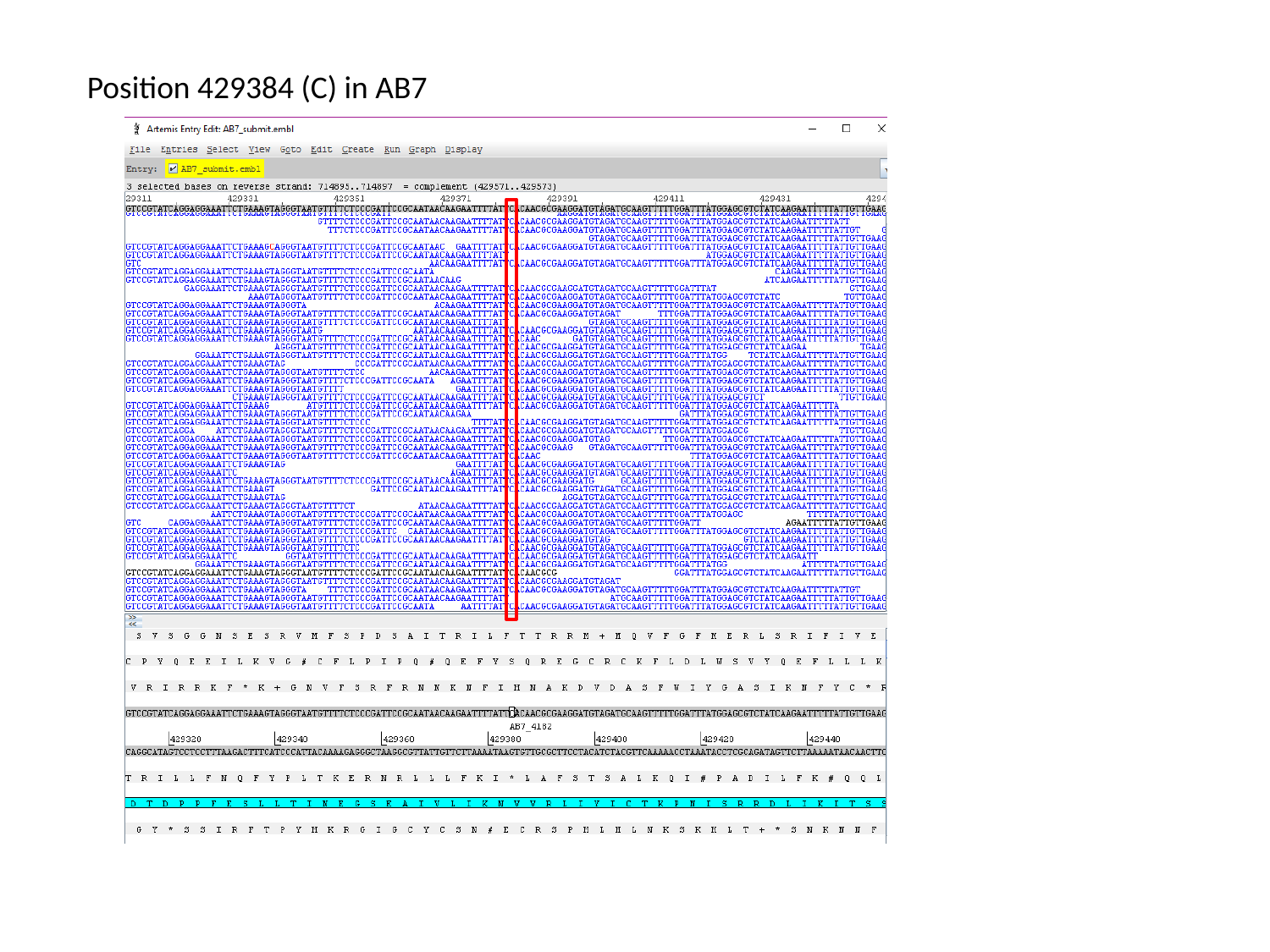

Position 429384 (C) in AB7

## Slide 26
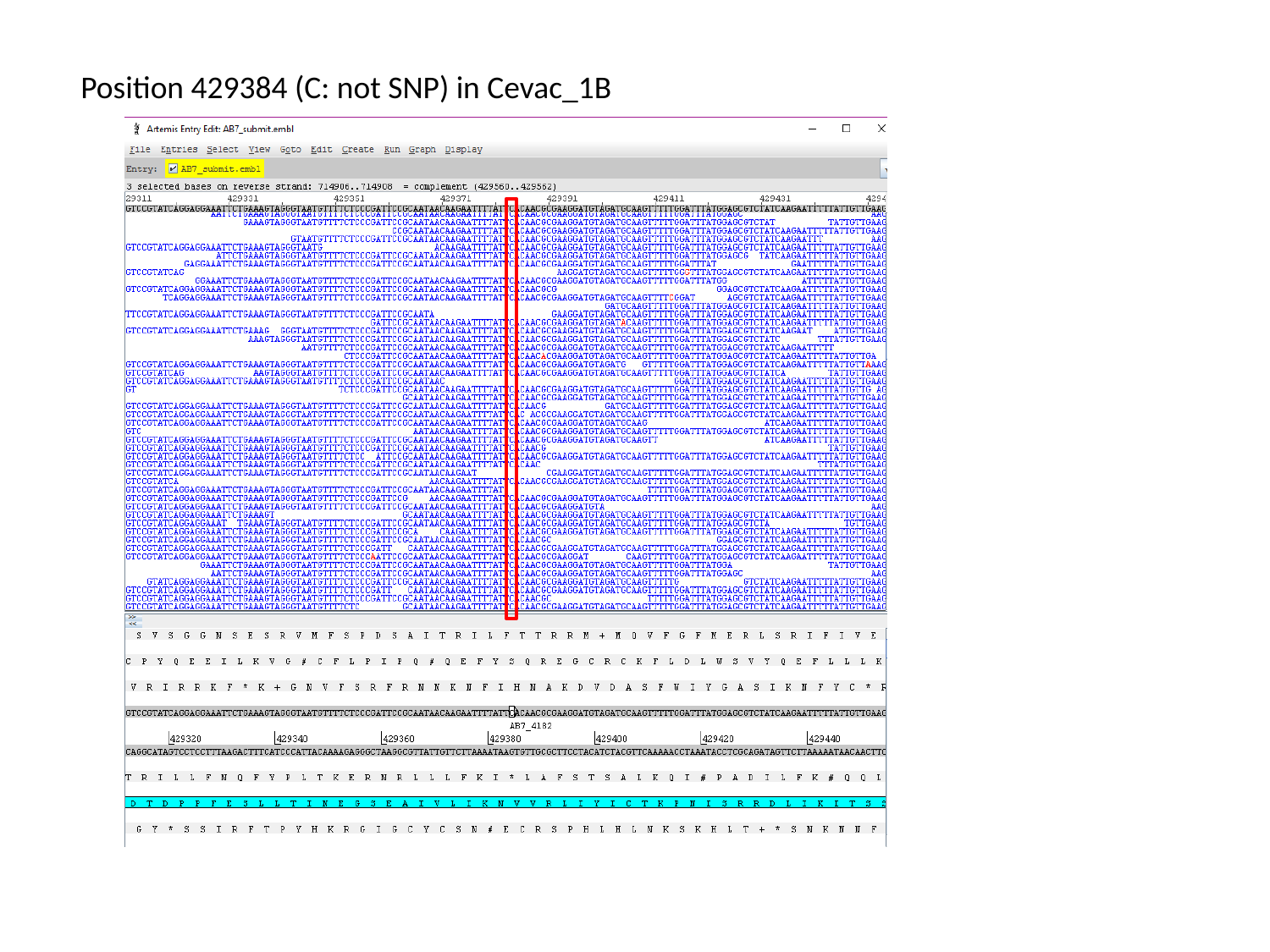

Position 429384 (C: not SNP) in Cevac_1B

## Slide 27
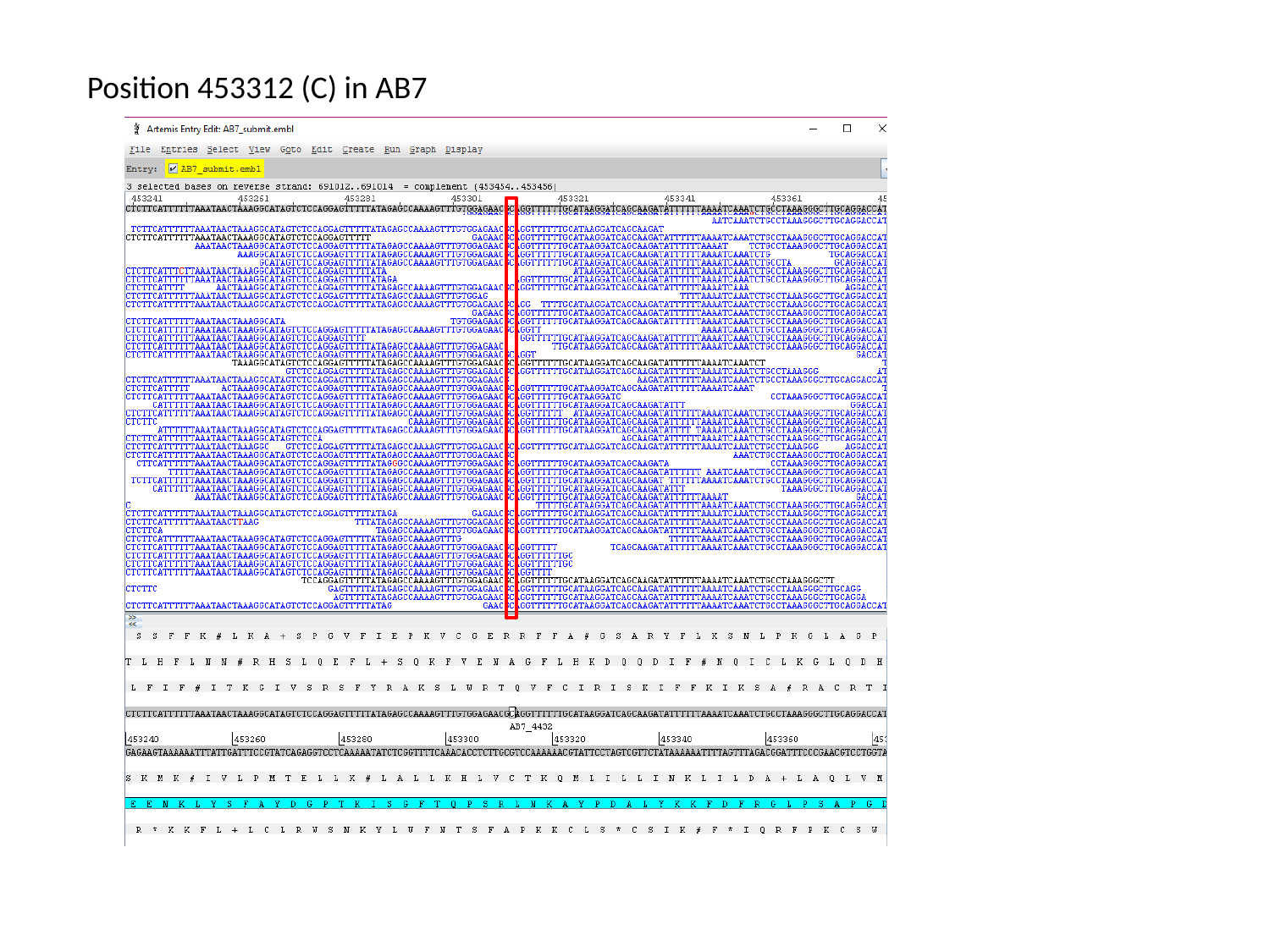

Position 453312 (C) in AB7

## Slide 28
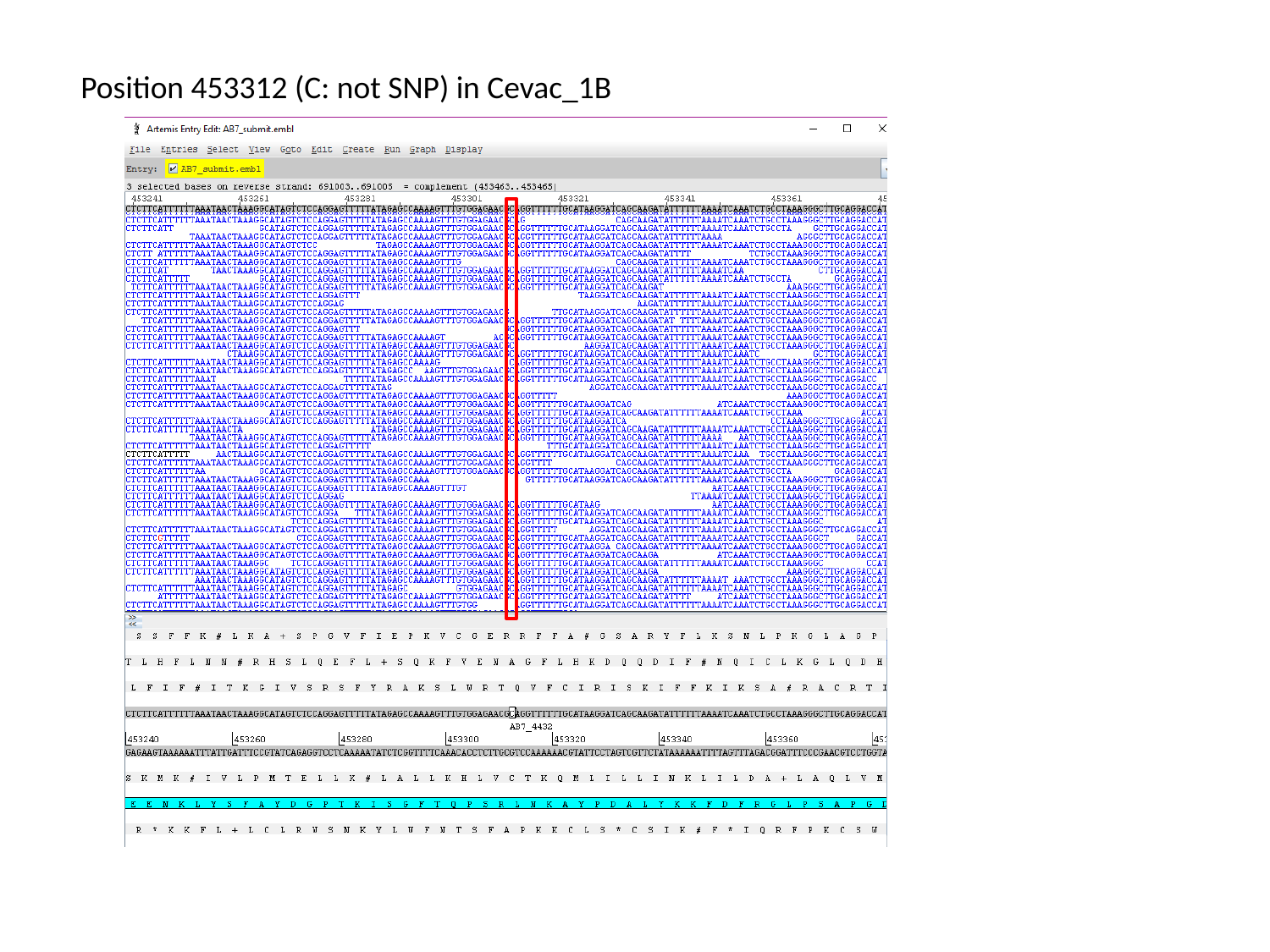

Position 453312 (C: not SNP) in Cevac_1B

## Slide 29
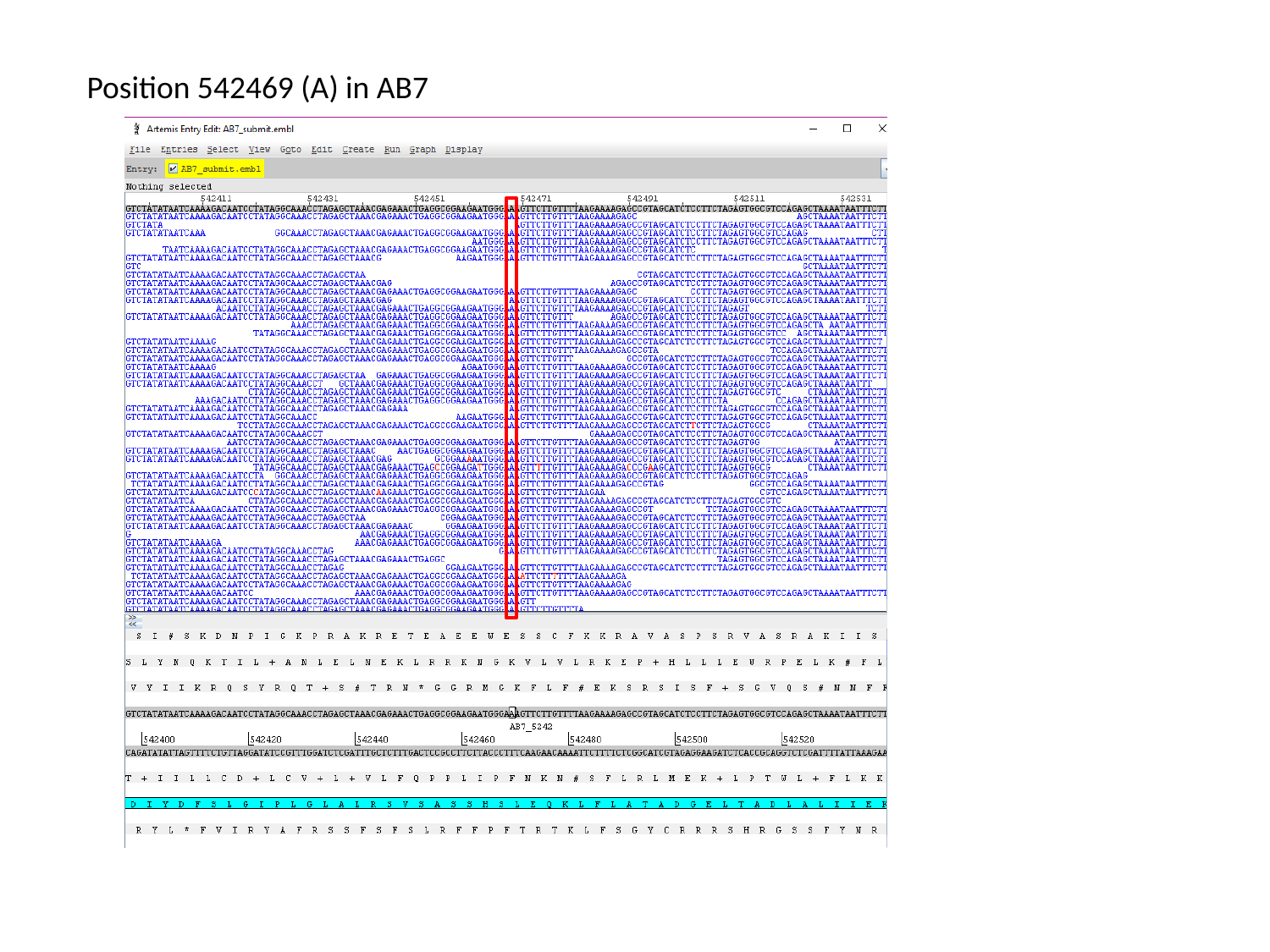

Position 542469 (A) in AB7

## Slide 30
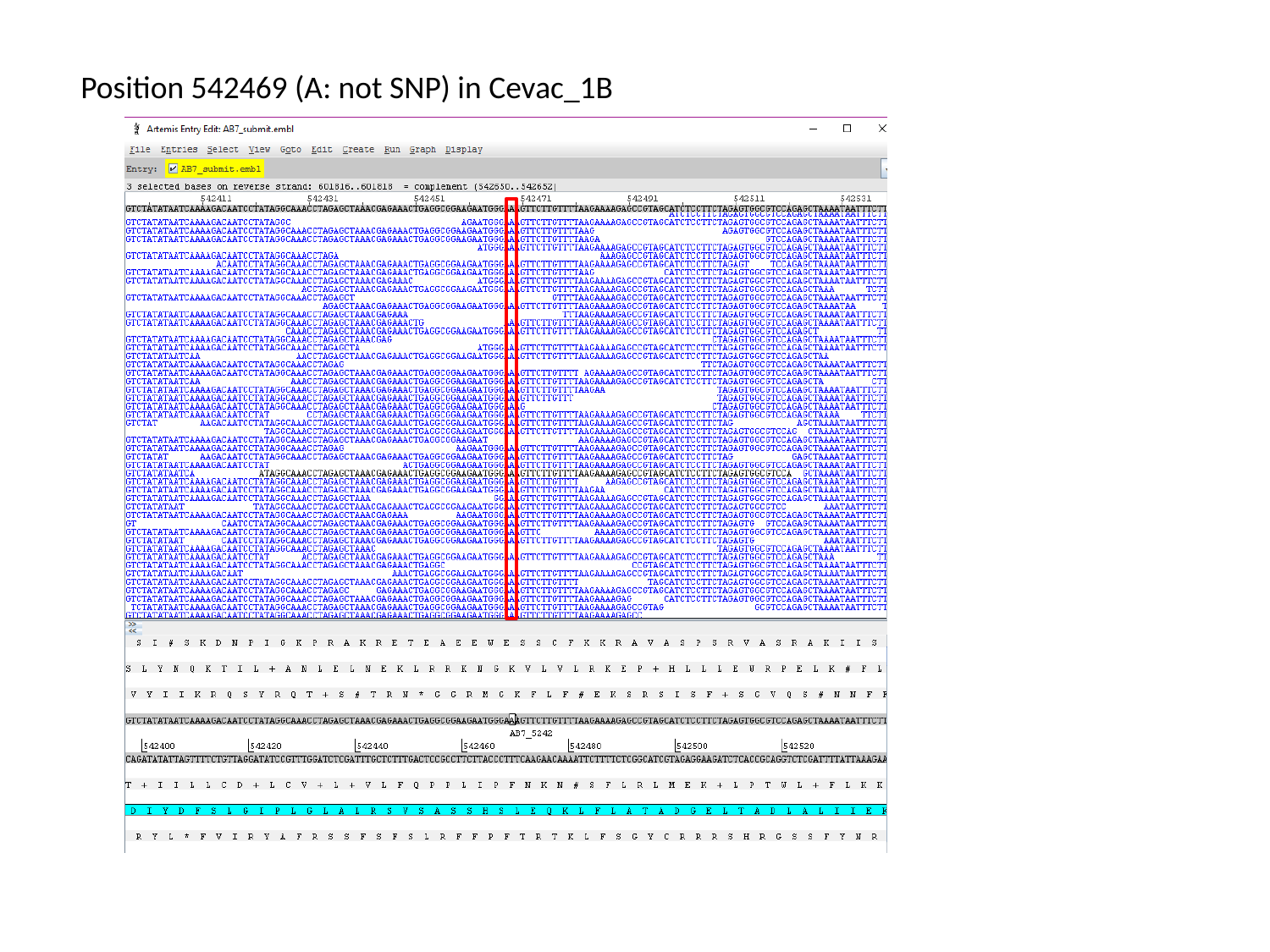

Position 542469 (A: not SNP) in Cevac_1B

## Slide 31
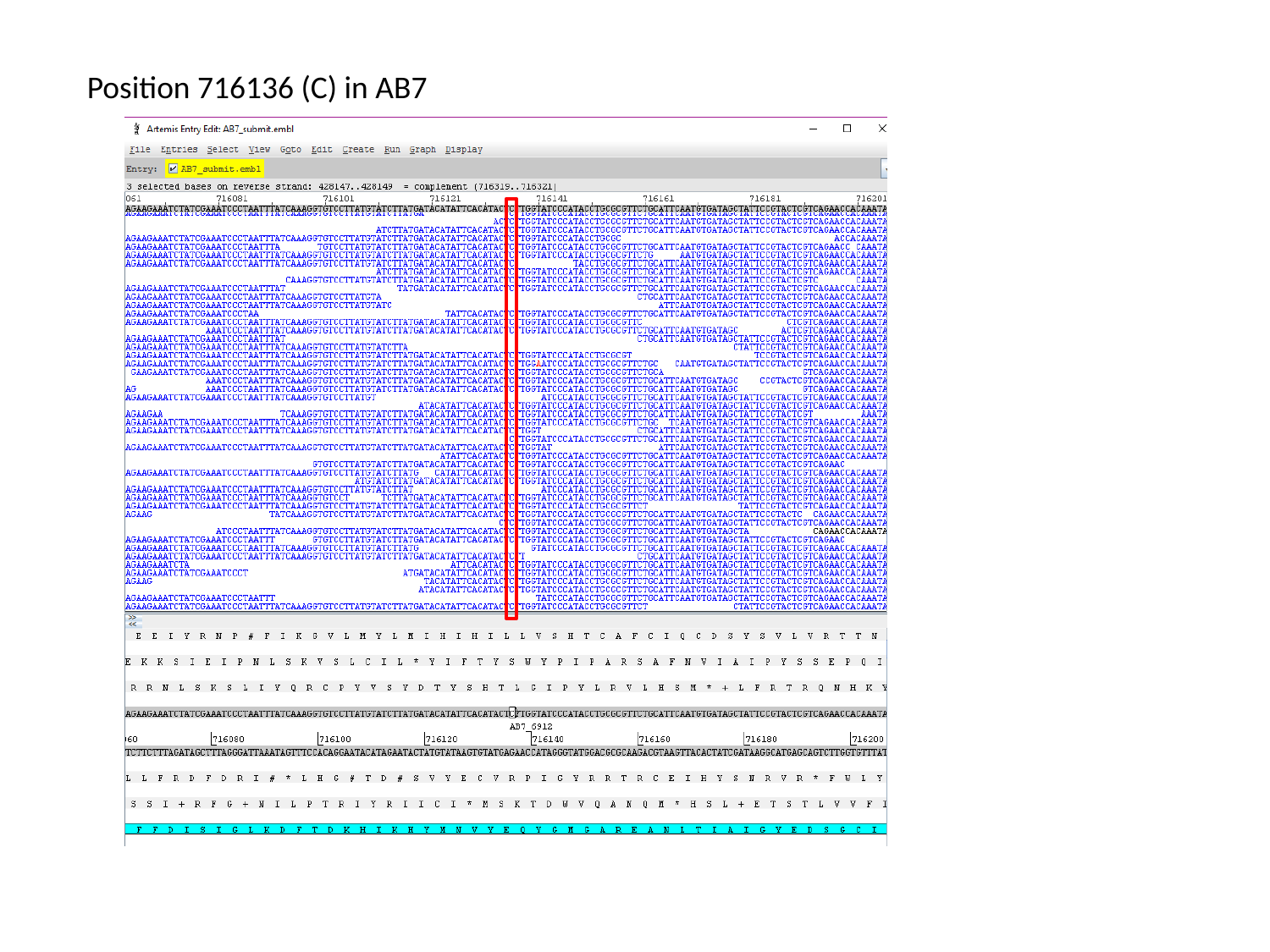

Position 716136 (C) in AB7

## Slide 32
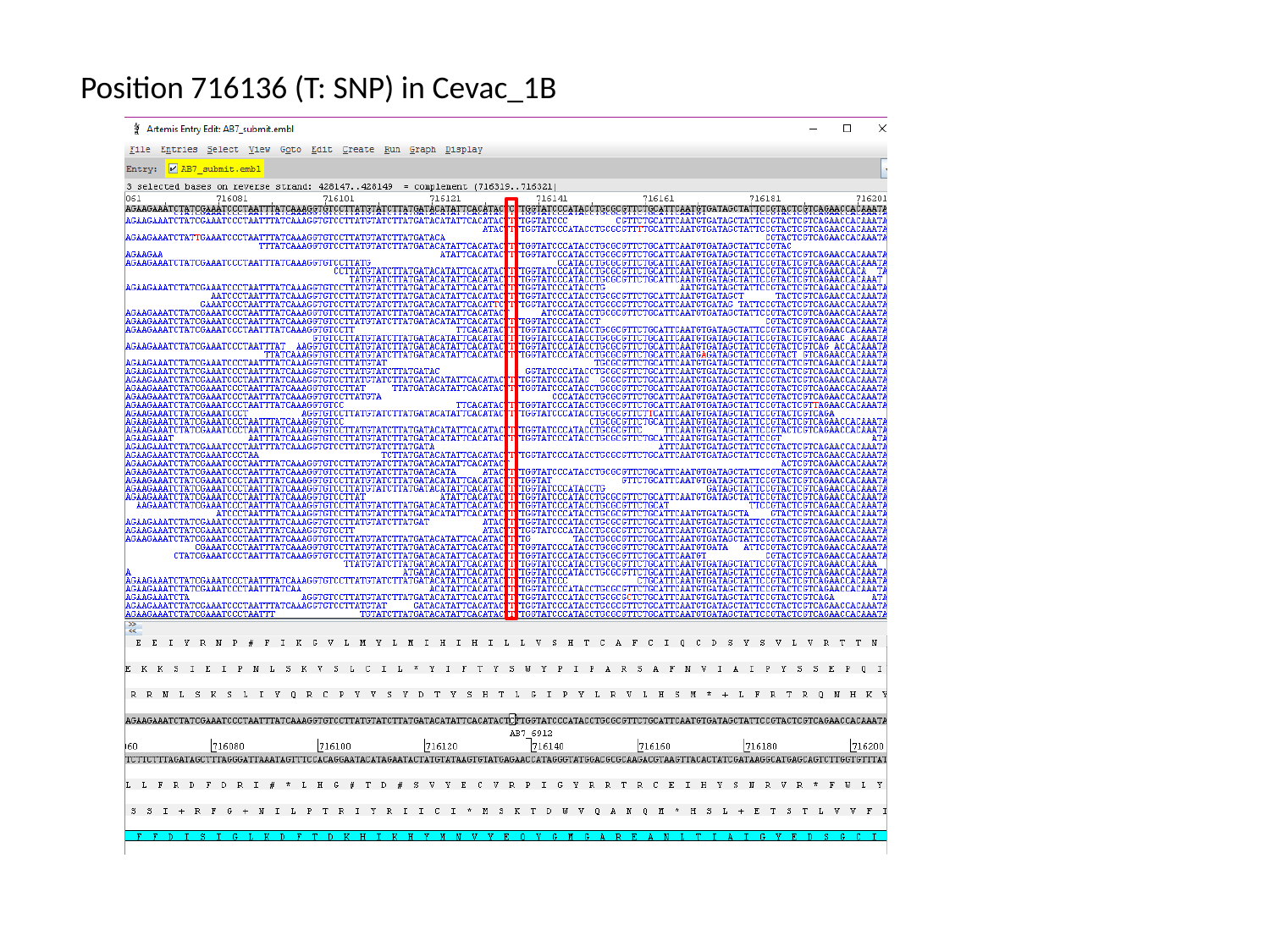

Position 716136 (T: SNP) in Cevac_1B

## Slide 33
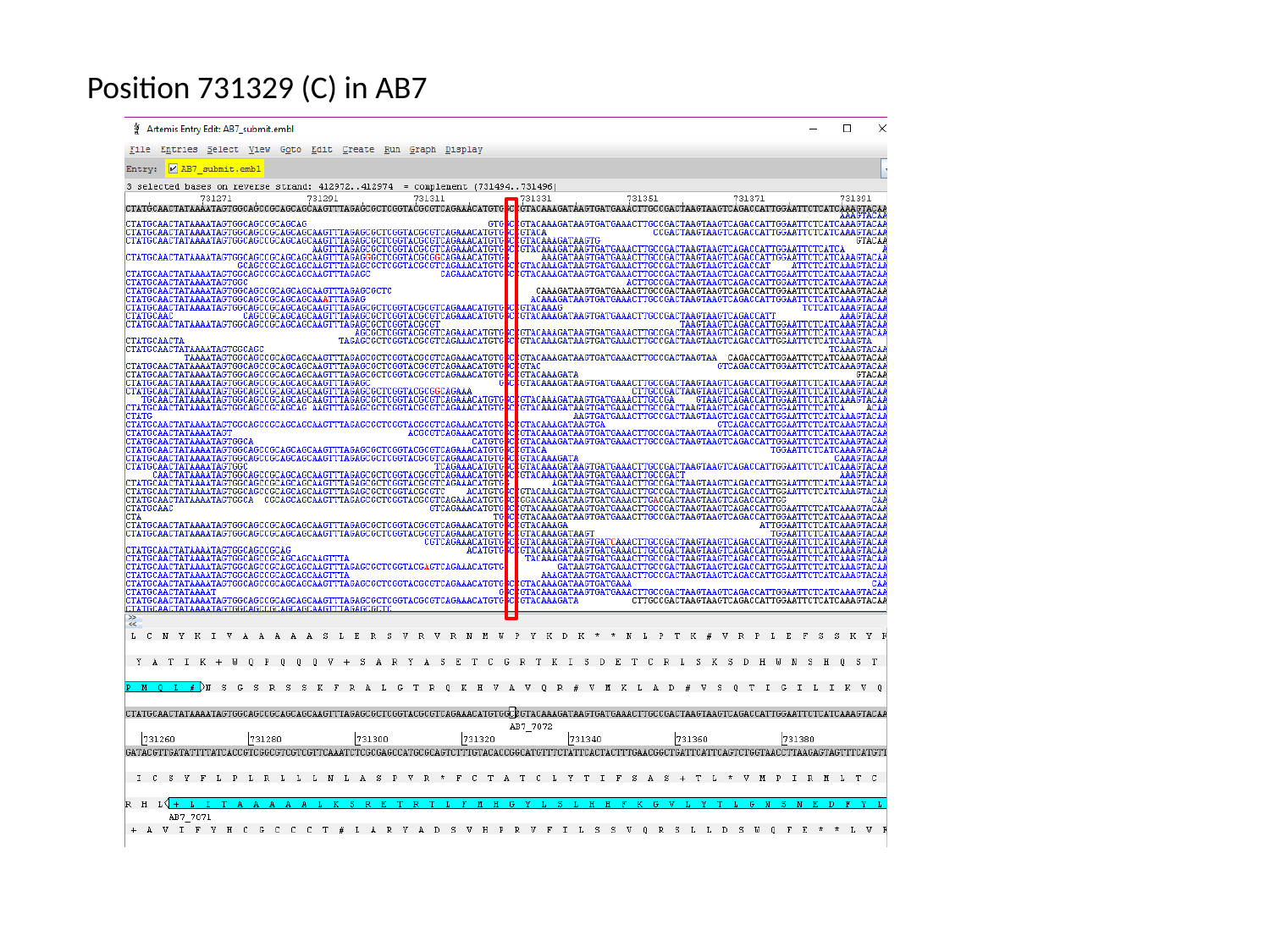

Position 731329 (C) in AB7

## Slide 34
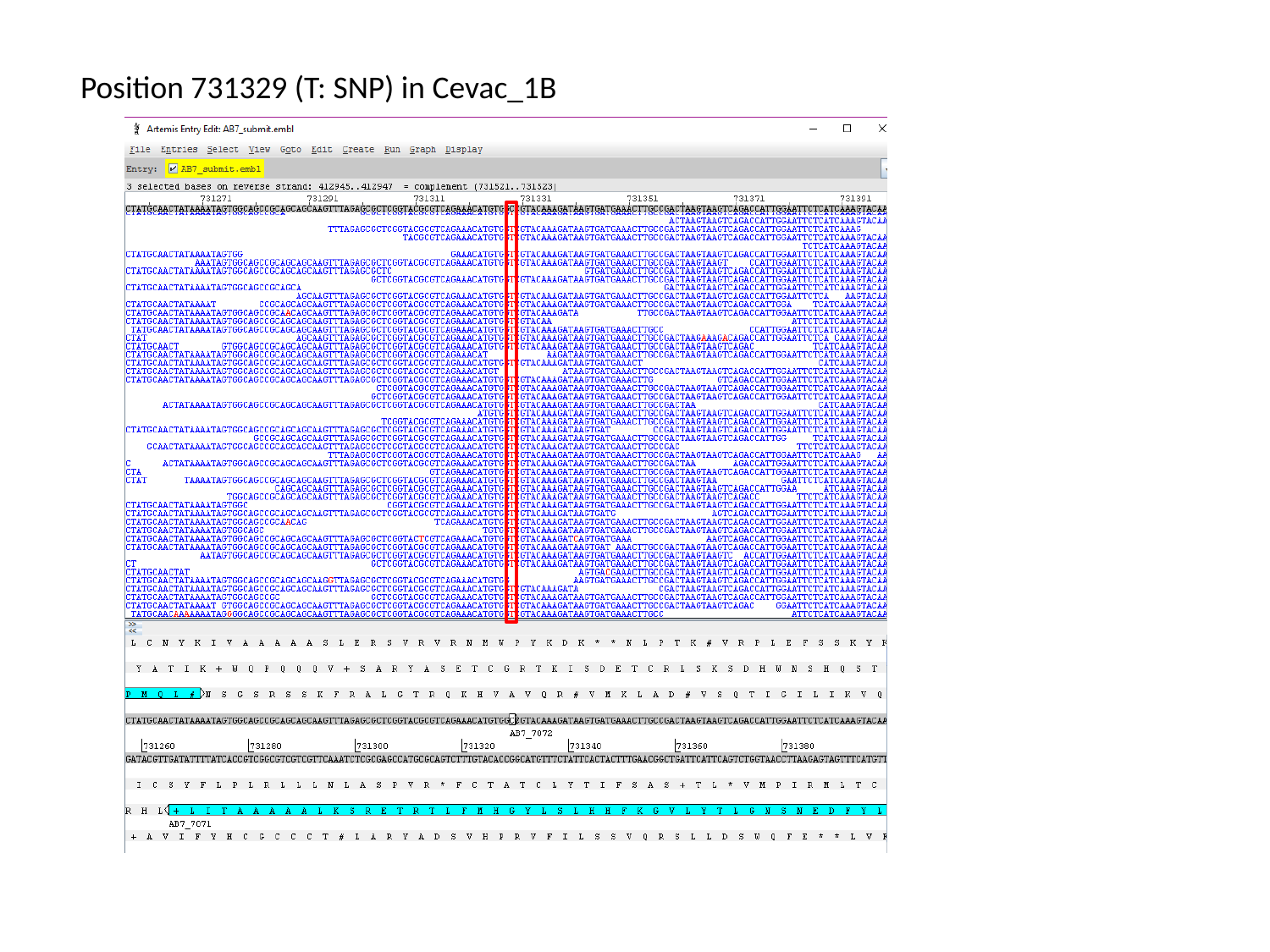

Position 731329 (T: SNP) in Cevac_1B

## Slide 35
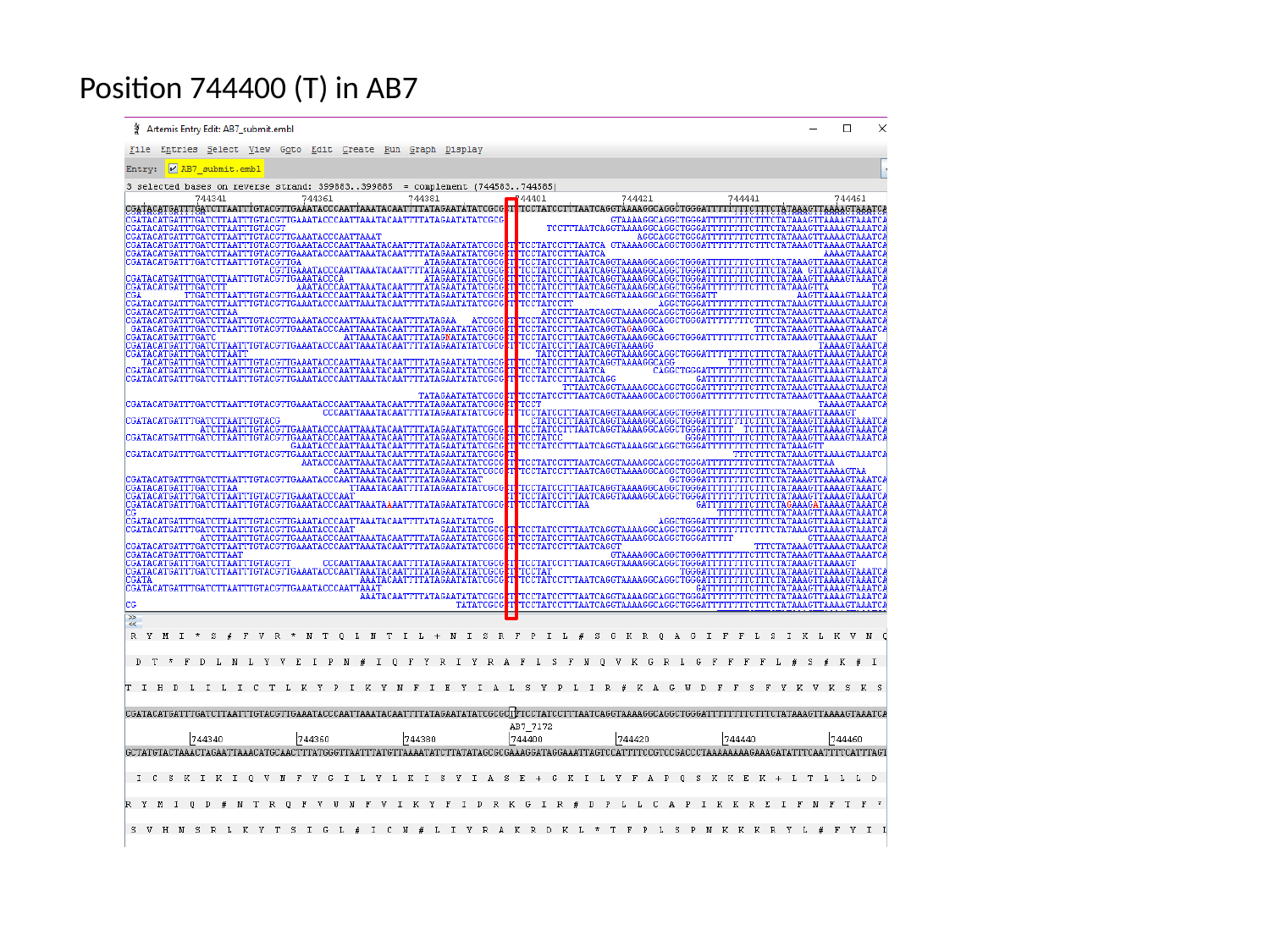

Position 744400 (T) in AB7

## Slide 36
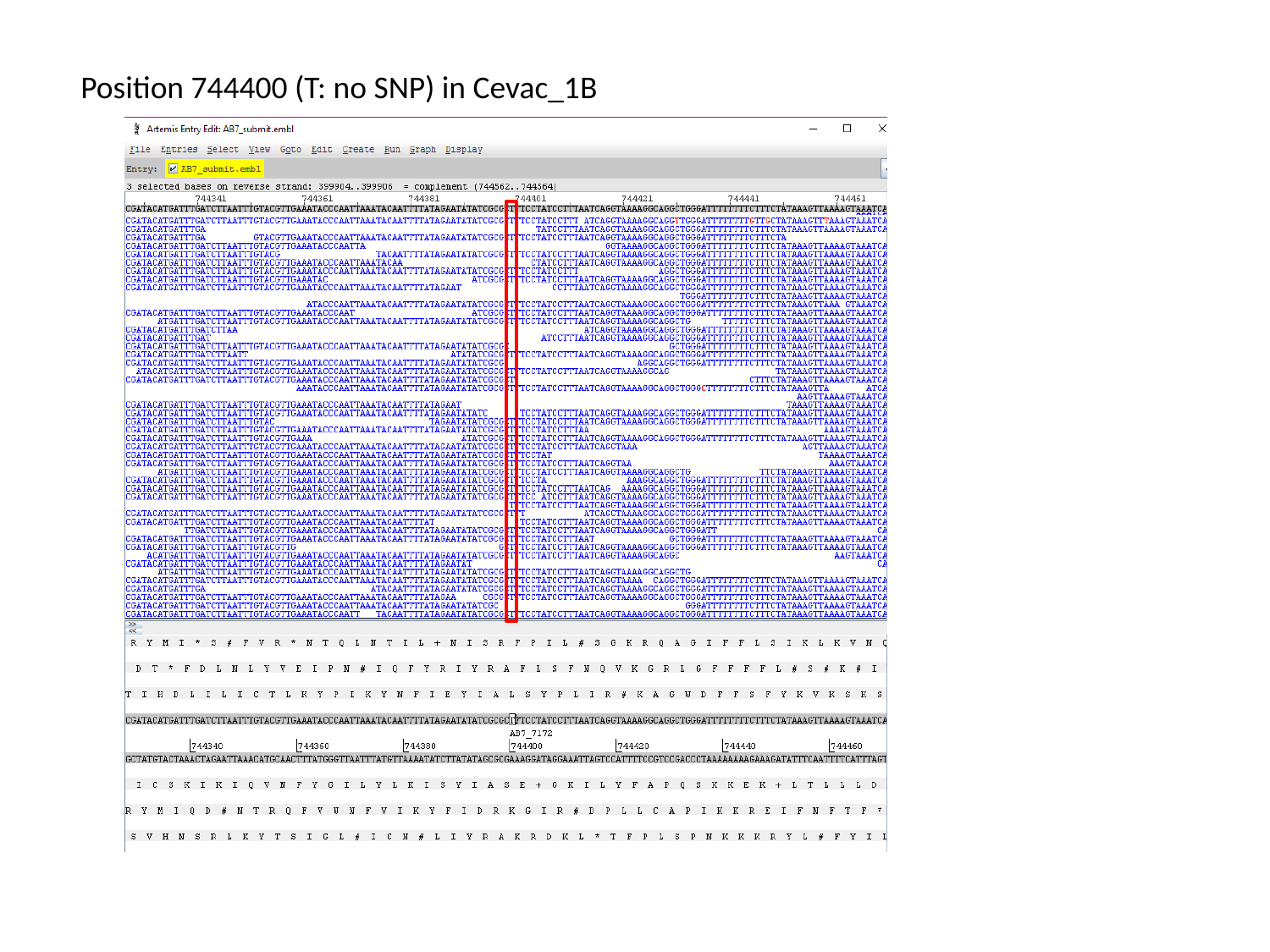

Position 744400 (T: no SNP) in Cevac_1B

## Slide 37
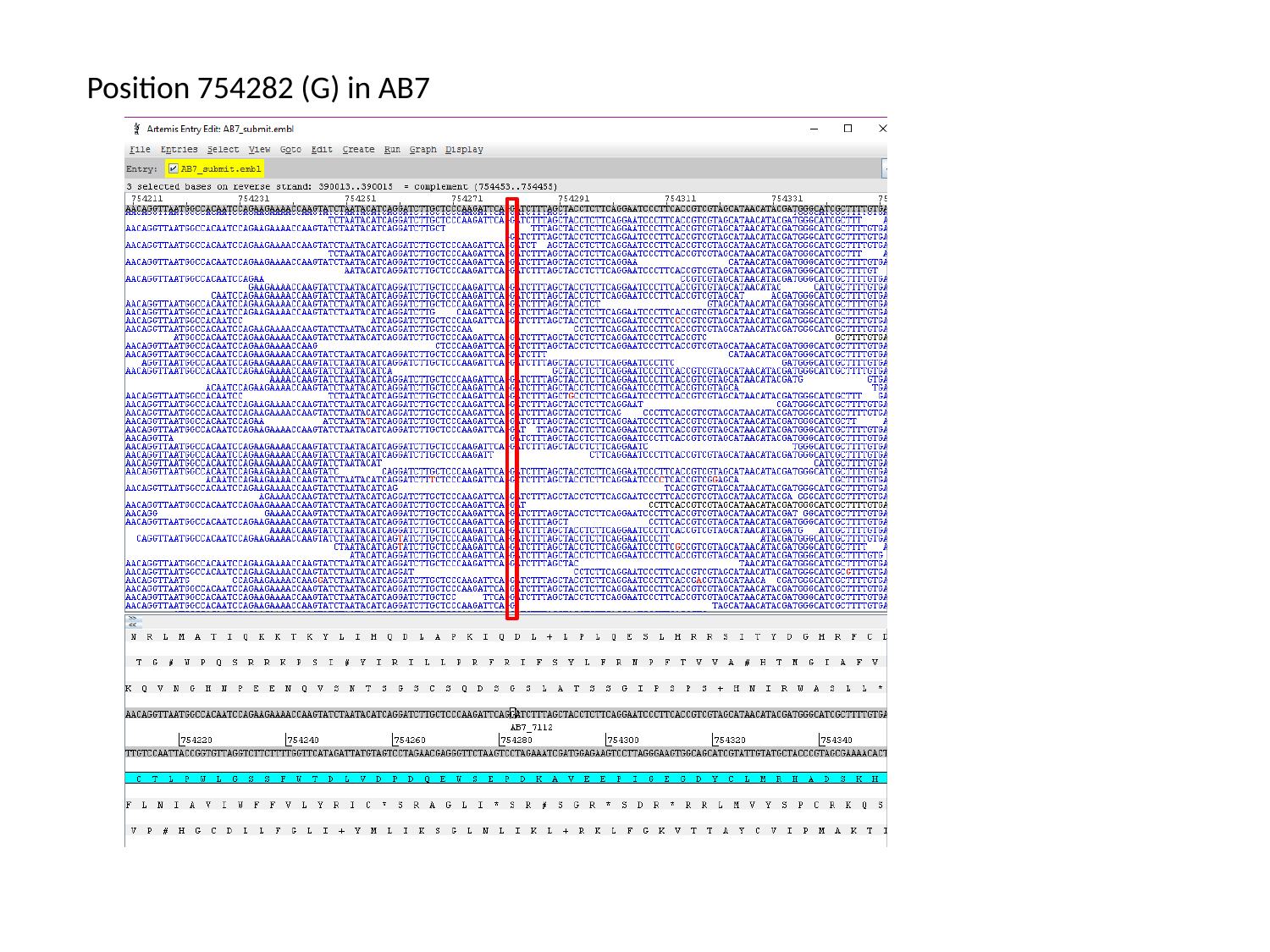

Position 754282 (G) in AB7

## Slide 38
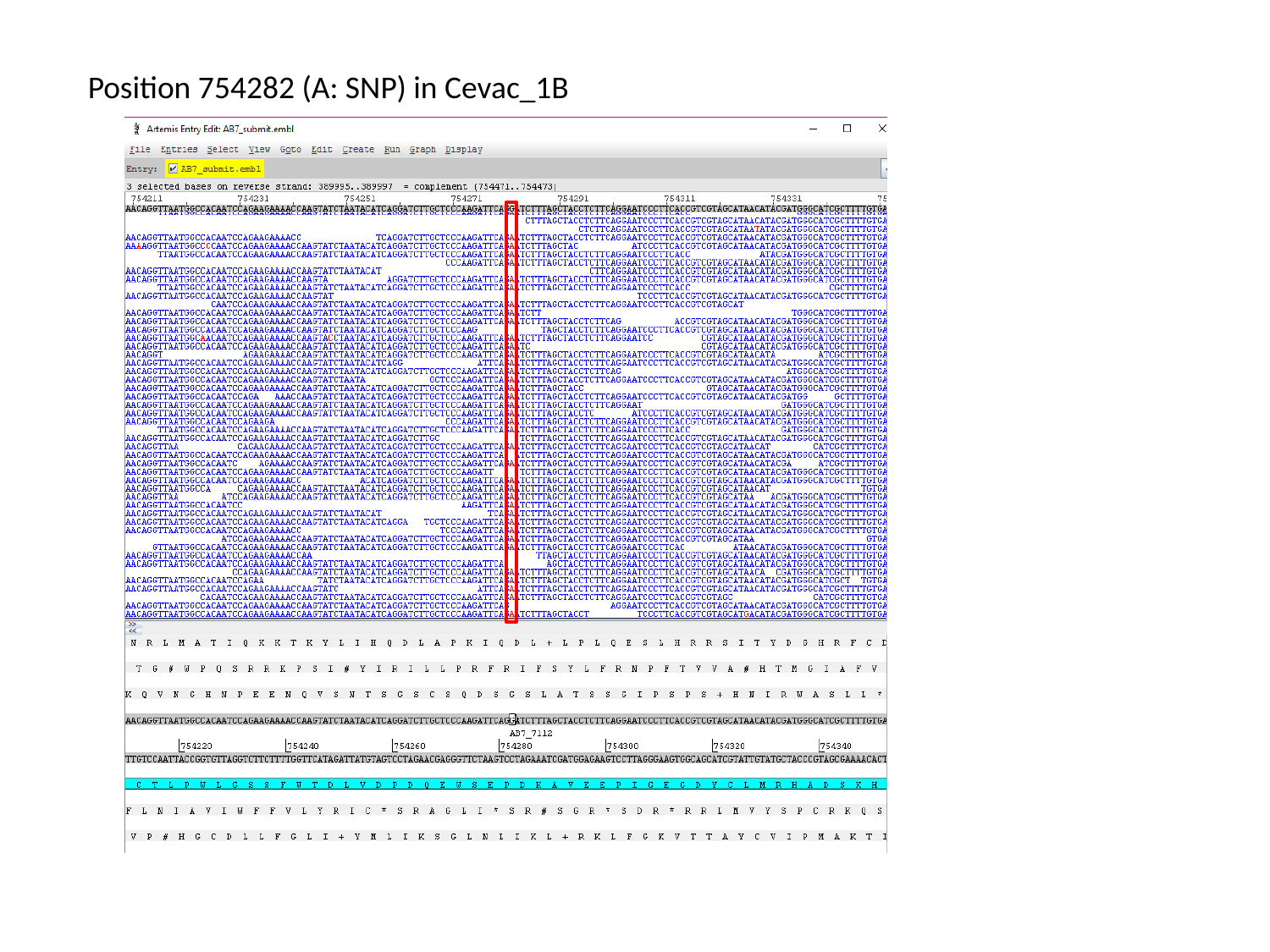

Position 754282 (A: SNP) in Cevac_1B

## Slide 39
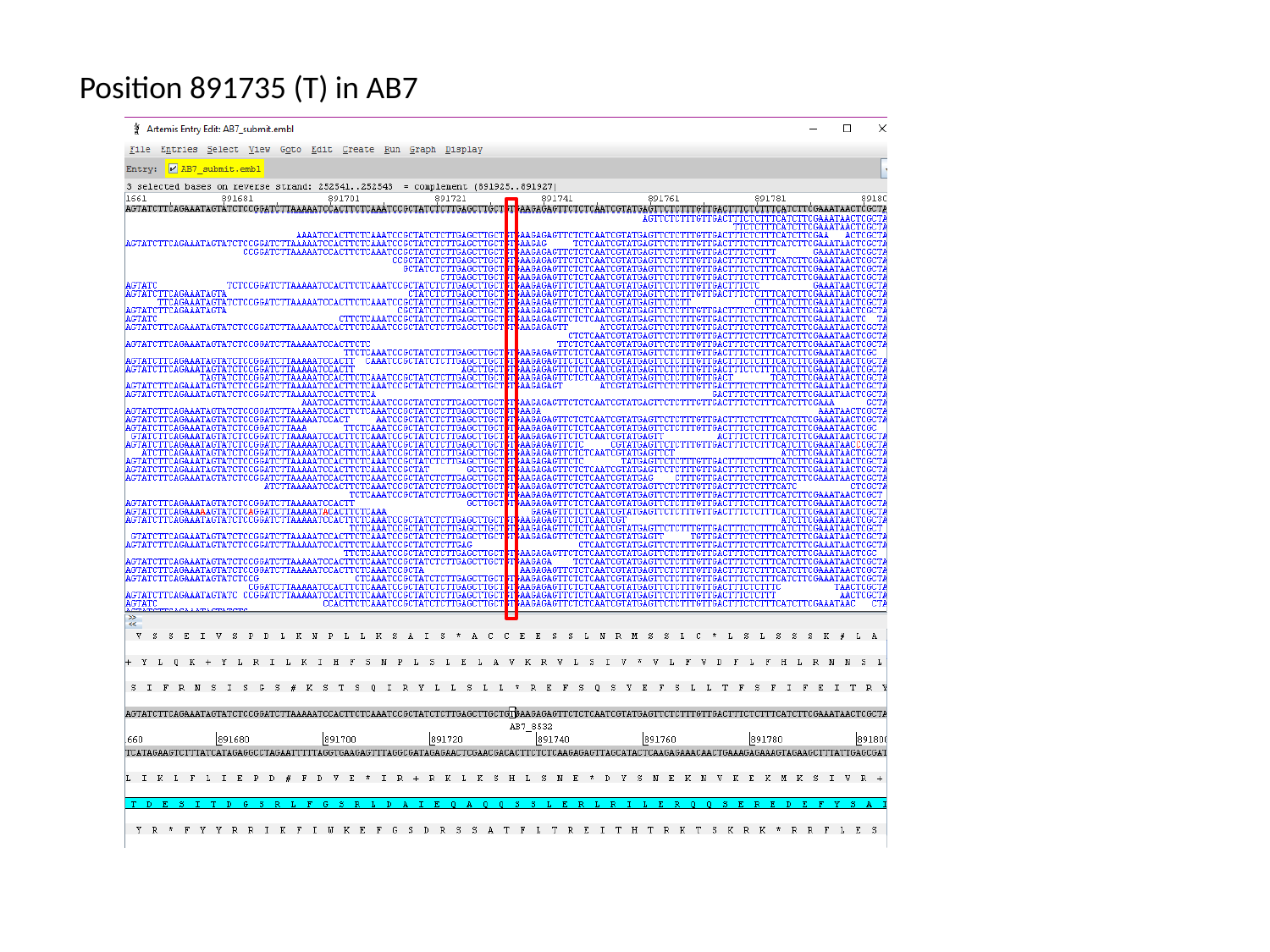

Position 891735 (T) in AB7

## Slide 40
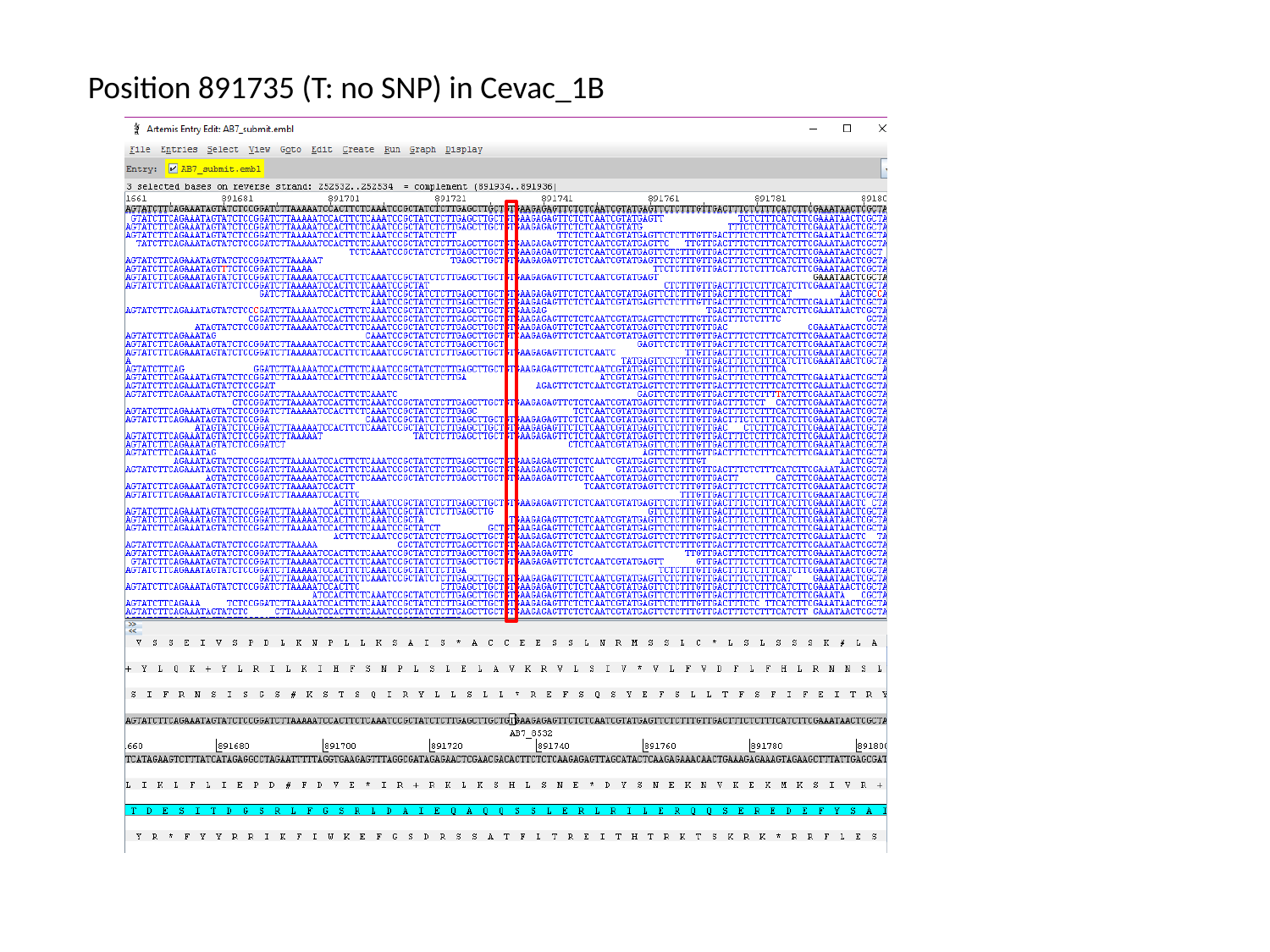

Position 891735 (T: no SNP) in Cevac_1B

## Slide 41
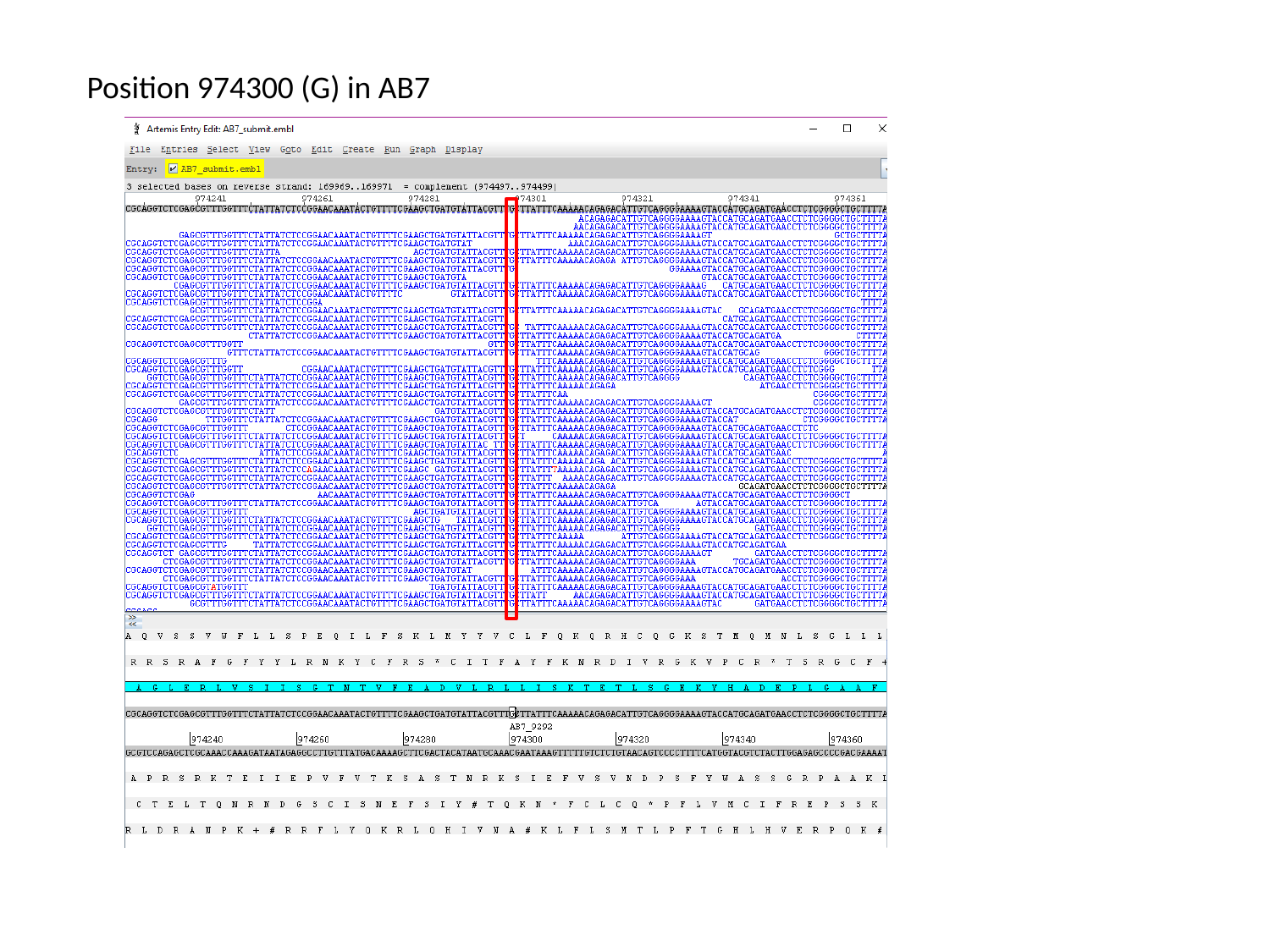

Position 974300 (G) in AB7

## Slide 42
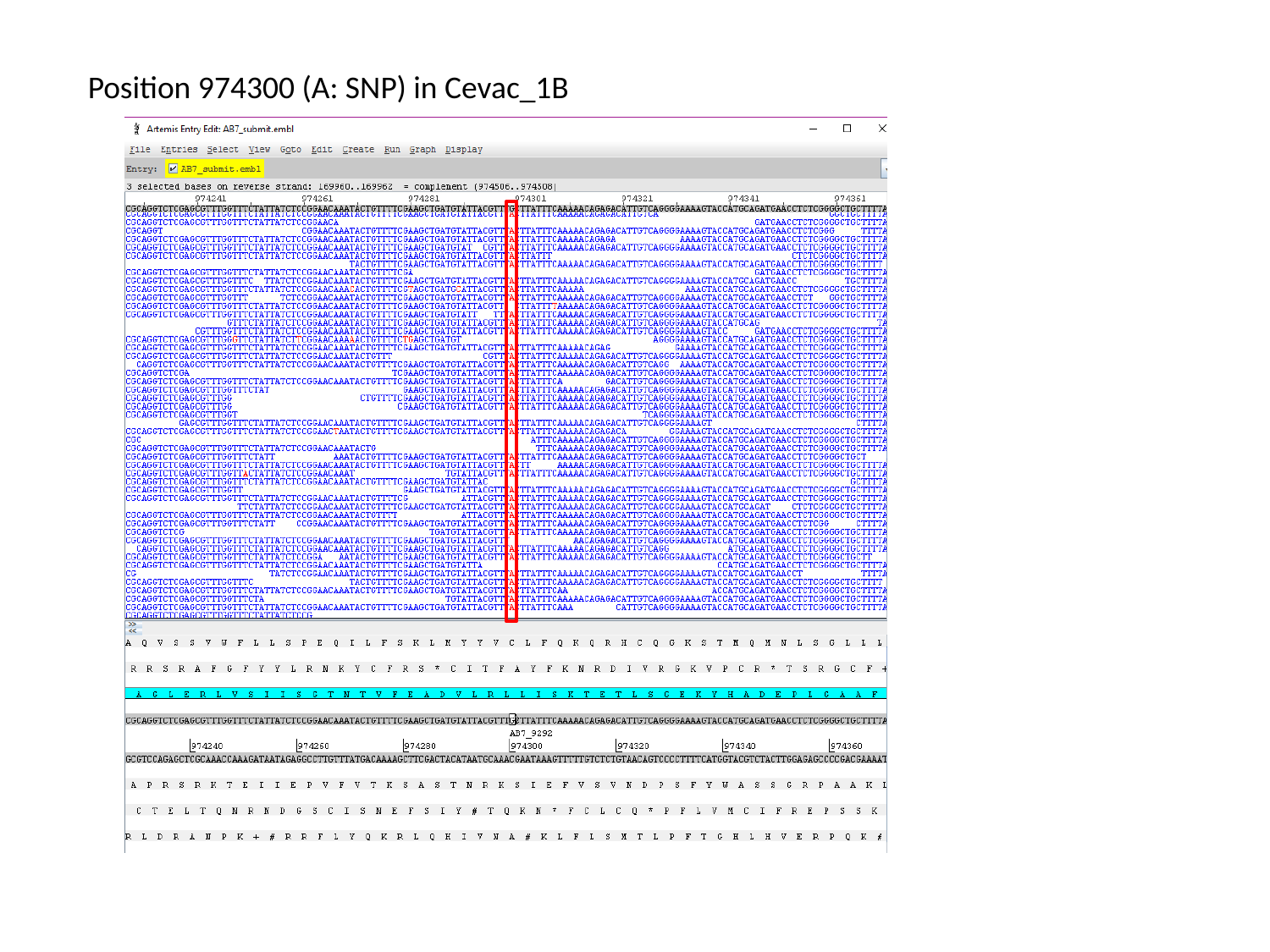

Position 974300 (A: SNP) in Cevac_1B

## Slide 43
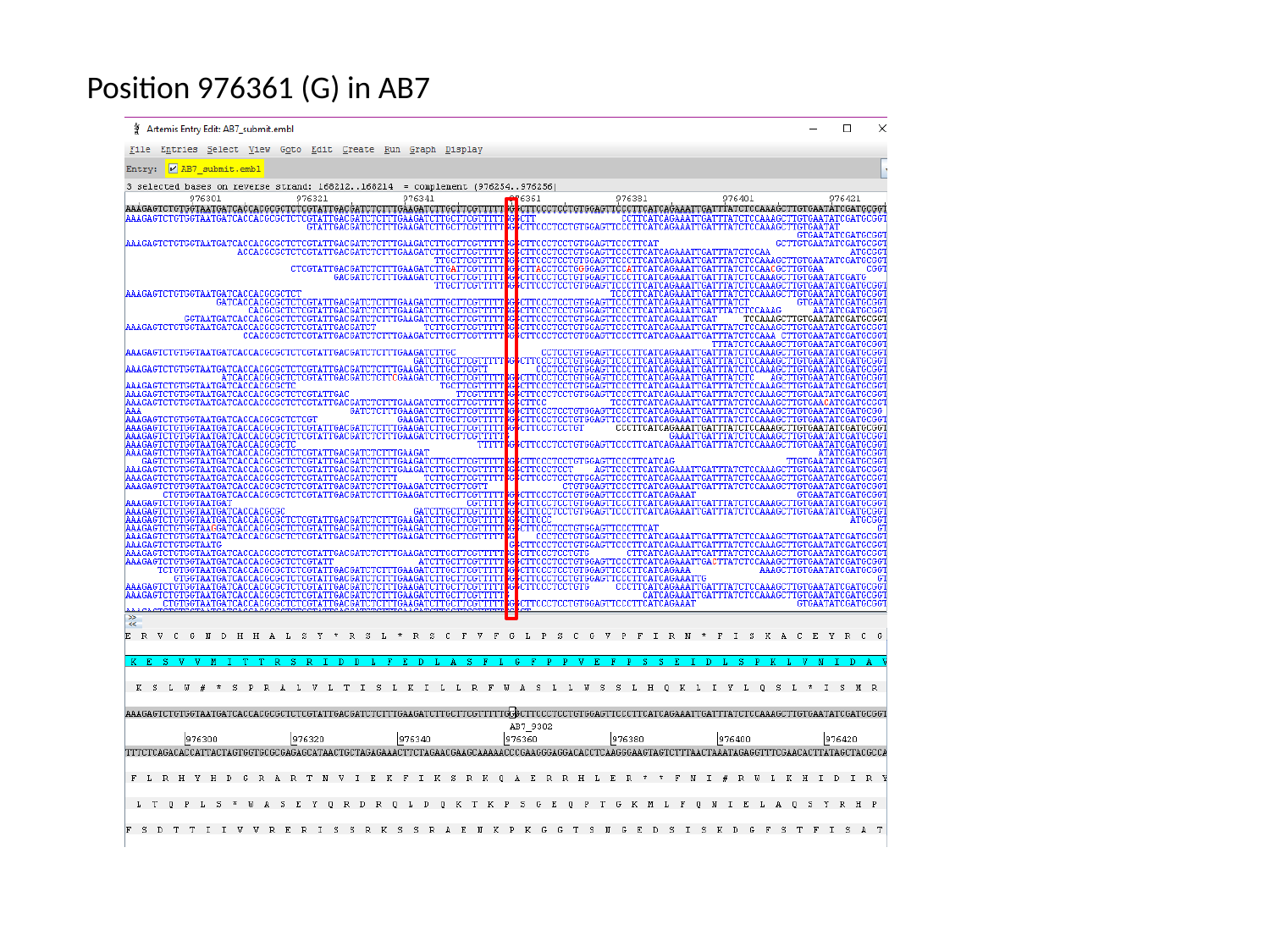

Position 976361 (G) in AB7

## Slide 44
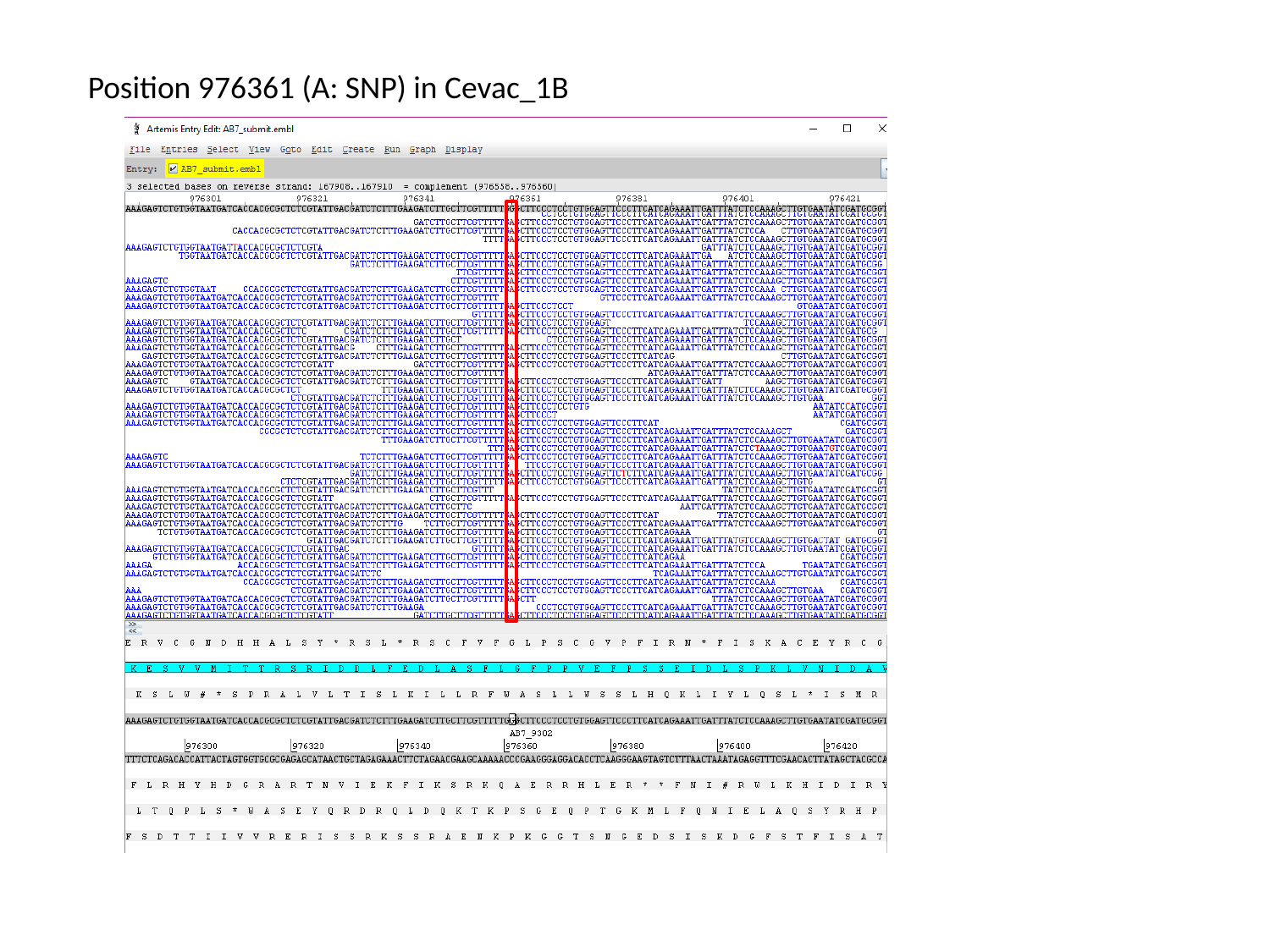

Position 976361 (A: SNP) in Cevac_1B

## Slide 45
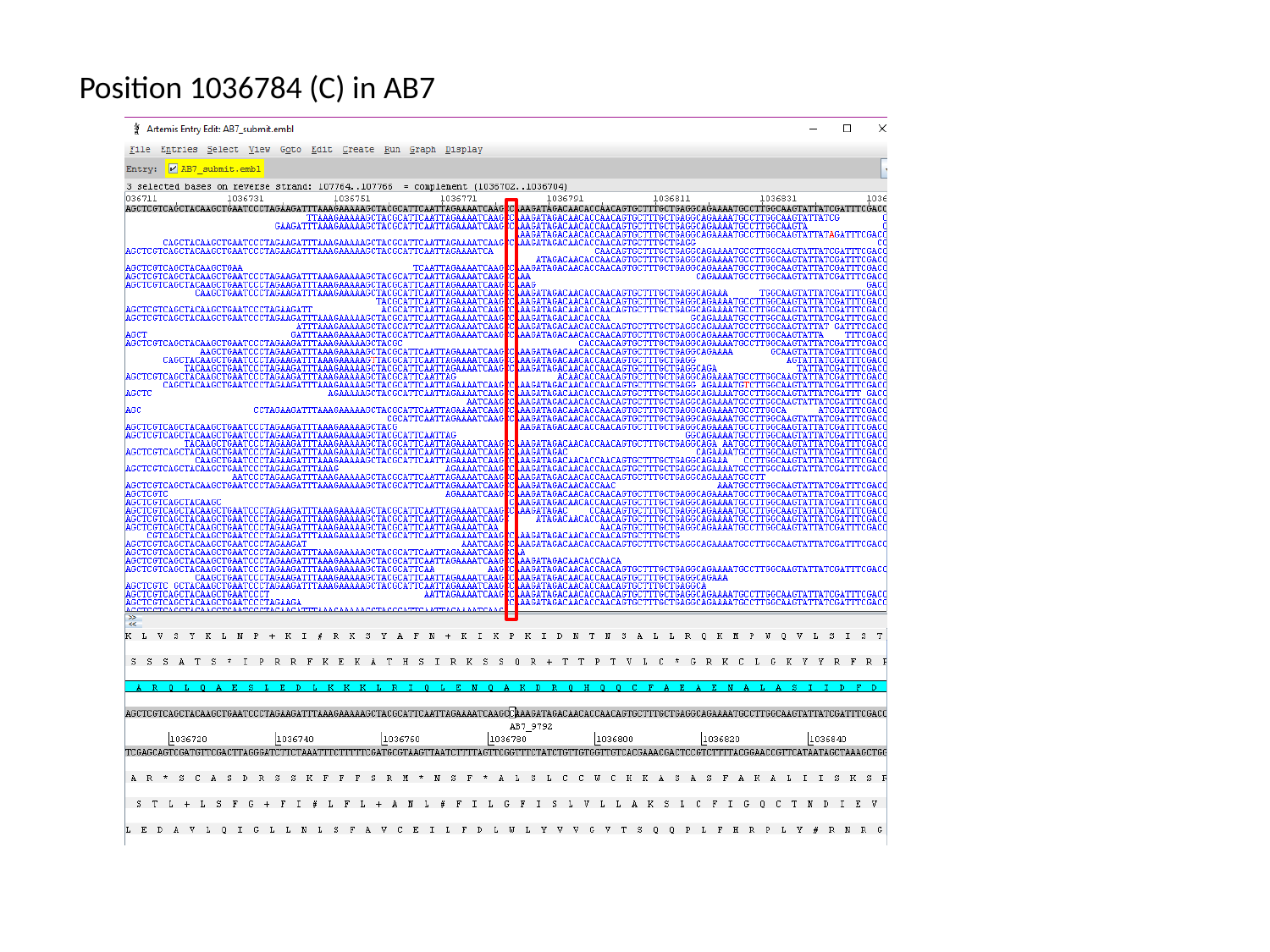

Position 1036784 (C) in AB7

## Slide 46
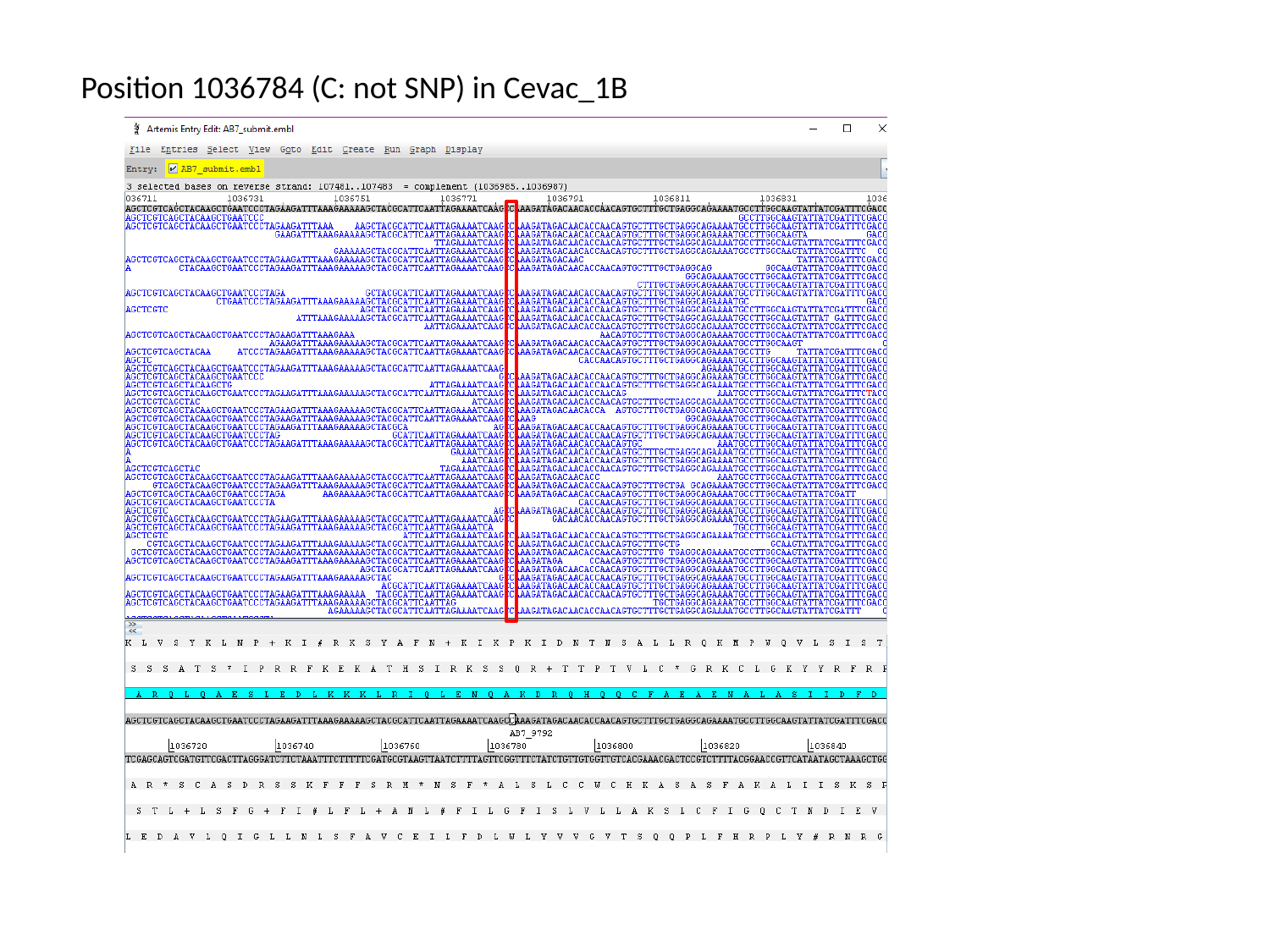

Position 1036784 (C: not SNP) in Cevac_1B
